# Supplementary material for: Cross-ancestry genome-wide analysis of atrial fibrillation unveils disease biology and enables cardioembolic risk prediction
Source: Nat Genet. 2023 Jan 19;55(2):187–97. doi: 10.1038/s41588-022-01284-9 (PMC9925380; doi:10.1038/s41588-022-01284-9)
Supplement: Supplementary file 1 — Supplementary Notes, Supplementary Methods, Supplementary references, List of Consortium Members and Supplementary Figs. 1–7. [file 41588_2022_1284_MOESM1_ESM.pdf]

nature genetics

Article

https://doi.org/10.1038/s41588-022-01284-9

Cross-ancestry genome-wide analysis of atrial fibrillation unveils disease biology and enables cardioembolic risk prediction

In the format provided by the authors and unedited

Supplementary Information

Table of Contents

Supplementary Notes *2*

Sex-stratified GWAS of AF 2

Shared allelic effects among populations 3

Credible set analysis 4

Pleiotropic effects of AF-associated loci 5

Functional pathway of AF-associated loci 5

Supplementary Methods *7*

Sex-stratified GWAS 7

Cross-ancestry genetic correlation 7

Credible set analysis 8

Pleiotropic analysis 9

Tissue and gene set enrichment analysis 9

References *11*

List of Consortium Members *14*

Supplementary Figures *17*

Supplementary Fig. 1 | Tissue enrichment analysis. 17

Supplementary Fig. 2 | Comparison of allele frequencies and allelic effects among ancestries and fine mapping derived from credible set analyses. 18

Supplementary Fig. 3 | Quantile-quantile plot for GWAS of three studies 20

Supplementary Fig. 4 | Sex-stratified GWAS. 21

Supplementary Fig. 5 | *CUX2* gene expression in liver. 23

Supplementary Fig. 6 | rs67329386 and transcription factor. 24

Supplementary Fig. 7 | Colocalization of quantitative traits with AF-associated signals. 25

Supplementary Notes

Sex-stratified GWAS of AF

Sex-related differences in the epidemiology, clinical presentation, and prognosis of AF have been consistently reported[1,2]. For example, the age-stratified prevalence of AF is lower in female than in male, despite a higher risk for AF-related outcomes such as stroke in female[3-5]. Thus, we divided our samples into males and females, and performed GWASs separately. First, to explore the difference in genetic susceptibility to AF between males and females, we calculated genetic correlation, which was 0.934 (s.e.m. 0.076) by GCTA-GREML[6] and 1.095 (s.e.m. 0.112) by LD-score regression[7], respectively. This result suggests that genetic influences on AF development are strongly shared between males and females. Next, we examined genetic loci with sex-dependent effects on AF development in the sex-stratified GWAS. We found 21 loci with genome-wide significance in the male GWAS, but only six loci in the female GWAS (Supplementary Fig. 4a and Supplementary Table 13). By comparing the effect sizes of the lead variants identified in the sex-stratified GWAS, we observed significant positive correlation and concordant allelic effects between sexes (Spearman's $\rho = 0.92$, $P = 7.4 \times 10^{-7}$; Supplementary Fig. 4b). On the other hand, we found genome-wide significant variants with a strong heterogeneity ($P_{het} < 1.0 \times 10^{-4}$) in the *PITX2-C4orf32* and the *CUX2* loci. The *PITX2-C4orf32* locus reached genome-wide significance in both sex-stratified GWAS (β_{male} (s.e.m.$_{male}$) = 0.569 (0.018), $P_{male} = 1.9 \times 10^{-210}$; β_{female} (s.e.m.$_{female}$) = 0.381 (0.027), $P_{female} = 3.0 \times 10^{-46}$, for rs12644625, the lead variant in the *PITX2-C4orf32* locus), while the *CUX2* locus in the female GWAS did not achieve genome-wide significance level (β_{male} (s.e.m.$_{male}$) = -0.280 (0.024), $P_{male} = 9.7 \times 10^{-33}$; β_{female} (s.e.m.$_{female}$) = -0.105 (0.034), $P_{female} = 2.1 \times 10^{-3}$, for rs3809297, the lead variant in the *CUX2* locus). Since the sample sizes in the sex-stratified GWAS were different (6,825 cases in the male-GWAS and 3,001 cases in the female-GWAS), we calculated the effective sample sizes with sufficient statistical power using the Genetic Association Study Power Calculator to examine whether the heterogeneity between sexes in these two loci was due to the difference in the sample sizes. To achieve 80% power, the effective sample size for the *PITX2-C4orf32* locus was approximately 450, while the *CUX2* locus required 3,000 cases. This

result shows that the sex-stratified GWAS has enough sample size with sufficient statistical power to detect the significant association signals of the two loci (Supplementary Table 14) and supports the genetic heterogeneity between sexes in the two loci rather than the mere difference in the sample sizes. Additionally, we performed an interaction analysis to investigate a SNP × sex effect, in which we performed an omnibus test for genome-wide significant variants with heterogeneity between sexes using regression models with or without the interaction term. As a result, we identified variants with a significant interaction effect with sex in the *PITX2-C4orf32* and *CUX2* loci (Supplementary Fig. 4c and Supplementary Table 15). Furthermore, *CUX2* is a transcription factor regulating sex-biased gene expression in rat and mouse liver[8,9], and the variant in the *CUX2* locus is associated with a DNA methylation site near the *ALDH2* gene, encoding aldehyde dehydrogenase, which catalyzes the oxidation of aldehydes into carboxylic acids[10]. According to Genotype-Tissue Expression (GTEx) data[11], *CUX2* gene expression in liver was significantly different between male and female ($P = 7.8 \times 10^{-4}$, Wilcoxon rank-sum test; Supplementary Fig. 5), suggesting that transcriptional disruption of sex-biased gene expression can lead to the difference in AF development between males and females through the metabolism of aldehydes in the liver. Thus, despite the small sample size in the female-GWAS to increase the false negative rate for association signals, the results from the interaction analysis and the different transcriptional regulation between sexes may provide evidence of the *PITX2-C4orf32* and the *CUX2* loci associated with gender difference in the genetic basis of AF.

Shared allelic effects among populations

We compared alternate allele frequencies and allelic effects for 150 lead variants between BBJ, EUR, and FIN. Compared to substantial concordance in allele frequencies between EUR and FIN (Spearman's $\rho = 0.974$, $P < 2.2 \times 10^{-16}$), we observed a moderate correlation in allele frequencies between BBJ and EUR as well as BBJ and FIN ($\rho = 0.592$, $P = 1.5 \times 10^{-15}$ and $\rho = 0.632$, $P < 2.2 \times 10^{-16}$, respectively; Supplementary Fig. 2a-c). Additionally, we found a significant positive correlation between concordant allelic effects of these variants ($\rho = 0.769$, $P < 2.2 \times 10^{-16}$

for BBJ vs. EUR; ρ = 0.769, $P < 2.2 \times 10^{-16}$ for BBJ vs. FIN; Supplementary Fig. 2d-f). To further explore the relationship of allelic effects between populations, we performed a cross-ancestry genetic correlation analysis, which showed strong correlation of BBJ with EUR and FIN (BBJ and EUR: $r_g = 0.990$, s.e.m. = 0.097, BBJ and FIN $r_g = 0.955$, s.e.m. = 0.344).

Credible set analysis

To assess the contribution of cross-ancestry meta-analyses to the refinement of the putative causal variants, we constructed 99% credible sets for the 150 AF-associated loci detected by the current cross-ancestry meta-analysis, and compared the number of variants included in the 99% credible sets derived from three combinations of the meta-analysis (EUR + BBJ, EUR + FIN, BBJ + EUR + FIN; Supplementary Fig. 2g, h). The size of the 99% credible sets derived from EUR + BBJ significantly decreased compared with those from EUR + FIN (*P* = 0.004, paired Wilcoxon rank-sum test). In addition, cross-ancestry meta-analysis of three datasets (BBJ + EUR + FIN) yielded the most significant decrease in the number of variants among all the combinations of meta-analyses (median number of variants = 12; interquartile range [IQR] 5 – 36; Supplementary Table 16). In particular, a single variant, rs67329386, was identified on the *ZFHX3* locus with a high posterior probability of 0.995, only in the cross-ancestry meta-analysis of three datasets. In previous AF-GWAS, several variants were found in the *ZFHX3* locus[12,13], where a large transcription factor, *ZFHX3*, together with *PITX2*, facilitated DNA binding and transcription activity[14]. To corroborate the role of rs67329386 as a causal variant, we searched for transcription factors binding to this locus using the ChIP-seq dataset in ChIP-Atlas[15]. Compared to other AF-associated variants within this locus, rs67329386 was distinctly located on the binding site of CEBPB (Supplementary Fig. 6), aiding in the recognition and binding of target gene regulatory regions, leading to cell proliferation, differentiation, immune response, and tumor formation[16].

Pleiotropic effects of AF-associated loci

To characterize the AF-associated loci, we examined the pleiotropic effects of the identified lead variants and proxies using the NHGRI-EBI GWAS catalog database. Of the 150 AF-associated loci, 112 (74.7%) had at least one overlapping variant, and we found 631 pairs of AF-associated variants and phenotypes, where blood pressure-related traits were most frequently observed (13.5%), followed by electrocardiogram-related (11.7%) and anthropometric traits (6.8%) (Supplementary Table 17). Given the heterogeneous study design and population in the GWAS catalog, we subsequently utilized the BBJ dataset[17-19] with a consistent study design and population, to investigate the pleotropic effects. In particular, to explore the biological pathways related to AF development, we assessed colocalization of AF-associated loci with quantitative trait loci (QTL) for clinical measurements such as anthropometric, metabolic, kidney-related, and blood pressure data. Colocalization analysis was performed per 150 AF-associated loci identified by the cross-ancestry meta-GWAS, and we identified 29 AF-associated loci that showed significant colocalization with QTL (Supplementary Fig. 7). Of 76 pairs of AF-associated loci and QTL with significant colocalization, 22.4% (17/76) were kidney-related traits, 15.8% (12/76) were blood pressure-related, 13.2% (10/76) were metabolic traits, and 13.2% (10/76) were anthropometric traits (Supplementary Table 18).

Functional pathway of AF-associated loci

To further enhance the biological understanding of the AF-associated loci, we performed in silico tissue and gene-set enrichment analysis using DEPICT software[20], which prioritizes candidate genes and tissues based on gene expression data from different tissues and cell types. Consistent with a previous study[21], we found significant enrichment of AF-associated loci predominantly in heart tissues ($P < 1.2 \times 10^{-5}$ for heart and $P < 2.3 \times 10^{-5}$ for heart atria; Supplementary Fig. 1 and Supplementary Table 19). Additionally, pathway analysis demonstrated that AF-associated loci were significantly enriched in 34 out of 1,157 gene sets, most of which were involved in heart development and morphogenesis (Supplementary Table 20). Among them, the

regulation of cell adhesion was a previously unreported pathway ($P = 1.6 \times 10^{-10}$), including *ZMIZ1* gene, the nearest for rs1769758, which was reported to regulate the activity of various transcription factors related to vascular development, androgen receptor coregulation, SMAD3 regulation, and coactivation of p53[22-24]. Further, it has been reported that knockdown of *ZMIZ1* in mice resulted in severe defects in the reorganization of the yolk sac vascular plexus and cell proliferation[25].

Supplementary Methods

Sex-stratified GWAS

In sex-stratified association analysis of 81,050 males (6,825 cases and 74,225 controls) and 69,222 females (3,001 cases and 66,221 controls), we also performed logistic regression analysis in males and females separately using the same pipeline as described above. Then, we performed the genetic correlation analysis using LD score regression[7]. As LD-score regression may estimate a genetic correlation beyond the range of -1 to 1 depending on sampling variation or disease heritability, we added a bivariate GREML analysis (http://cnsgenomics.com/software/gcta/#BivariateGREMLanalysis) implemented in GCTA software[6]. Next, we directly compared effect sizes of the variants identified in the sex-stratified GWAS. To calculate the effective sample sizes with sufficient statistical power, we used the Genetic Association Study Power Calculator provided by the University of Michigan (https://csg.sph.umich.edu/abecasis/gas_power_calculator/), under the following conditions: a significance level of $P < 5 \times 10^{-8}$ and disease prevalence in the Japanese population of 0.82% (https://vizhub.healthdata.org/gbd-compare/). We also used the allele frequencies and genotype relative risks of the lead variants in the *PITX2-C4orf32* and *CUX2* loci as an input data (Supplementary Table 14). The effective sample size was defined as the minimum number of samples to achieve 80% power. In the interaction analysis, we tested for the interaction effect of a SNP × sex on AF development, where we performed an omnibus test using models with and without the interaction term. Chi-square statistics were calculated from the omnibus test. Since limiting to variants with a strong heterogeneity ($P_{het} < 0.0001$) may lead to some association signals being missed, we analyzed genome-wide significant variants with a relaxed heterogeneity threshold ($P < 0.05$).

Cross-ancestry genetic correlation

To estimate the cross-ancestry genetic correlation, we applied Popcorn software (v.1.0). We used East Asia or European samples of 1KG for the LD reference and excluded the variants

within the major histocompatibility complex region according to the instructions of the software. To estimate the liability-scale genetic correlation, we assumed that the AF prevalence was 0.58% in the Japanese population, 1.38% in the United Kingdom population, and 0.97% in the Finnish population (https://vizhub.healthdata.org/gbd-compare/).

Credible set analysis

To identify sets of variants that likely include causal variants, we constructed a 99% credible set per 150 AF-associated loci identified by the cross-ancestry meta-analysis. For each locus, we calculated the posterior probability (PP) for the jth SNP using BF obtained from MANTRA results in the cross-ancestry meta-analysis and the following formula: $PP_j = \frac{BF_j}{\sum_k BF_k}$, where BF_j denotes the BF for the jth SNP and BF_k denotes all of the variants included in the locus. We then constructed the 99% credible set by adding the variants in the order of decreasing PP, starting from a lead variant, where the sum of PP included in the region is 99%. To assess whether the 99% credible set derived from the cross-ancestry meta-analysis narrowed down the causal variants, we performed two additional meta-analyses (EUR + BBJ and EUR + FIN) using the MANTRA algorithm and constructed 99% credible sets for 150 AF-associated loci identified by the cross-ancestry meta-analysis. When we compared the fine-mapping effect of cross-ancestry meta-analysis, the sample size in FIN + BBJ (n = 213,894) was much smaller than those in the other combinations (EUR + BBJ; n = 1,181,108, EUR + FIN; n = 1,094,458, and BBJ + EUR + FIN; n = 1,244,730), which may lead to difficulty in distinguishing the sample size effect from the fine-mapping effect of a cross-ancestry meta-analysis. Accordingly, we examined the effect of a cross-ancestry meta-analysis by comparing three meta-GWASs (EUR + BBJ, EUR + FIN, and BBJ + EUR + FIN). We then compared the number of variants in the 99% credible set for each locus. Among them, the loci where at least one variant surpassed genome-wide significance ($\log_{10}$ BF > 6) in all meta-analyses were included in the test. The differences in the sizes of the credible sets were tested using the paired Wilcoxon rank-sum test.

Pleiotropic analysis

To assess the pleiotropic effects of AF-associated variants, we selected the lead variants and their proxies with $r^2 > 0.8$ across 150 AF-associated loci from the cross-ancestry meta-analysis, and searched if these variants overlapped with other diseases or traits using NHGRI-EBI GWAS catalog on February 8, 2019.

For colocalization analysis, we used the summary statistics of QTL from the BBJ dataset[17-19] for clinical parameters of nine distinct categories; anthropometric (height and body mass index), metabolic (total cholesterol, high-density-lipoprotein cholesterol, low-density-lipoprotein cholesterol, triglyceride, blood sugar, and hemoglobin A1c), serum protein (total protein and albumin), kidney-related (blood urea nitrogen, serum creatinine, estimated glomerular filtration rate, and uric acid), electrolyte (sodium, potassium, and chloride), liver-related (total bilirubin, aspartate aminotransferase, alanine aminotransferase, alkaline phosphatase, and γ-glutamyl transferase), other biochemical (activated partial thromboplastin time, prothrombin time, creatine kinase, lactate dehydrogenase, and C-reactive protein), hematological (white blood cell count, red blood cell count, hemoglobin, hematocrit, and platelet), and blood pressure (systolic blood pressure, diastolic blood pressure, mean arterial pressure, and pulse pressure). We extracted QTL data based on 150 AF-associated loci identified by the cross-ancestry meta-GWAS, and colocalization was performed for each locus using Coloc.abf from the Coloc R package[26]. The thresholds of coloc $H3 + H4 \geq 0.8$ and $H4/H3 \geq 2$ were applied for significant colocalization by referring to a prior study[27].

Tissue and gene set enrichment analysis

For tissue and gene set enrichment analysis, we used data-driven expression-prioritized integration for complex traits (DEPICT) software (https://data.broadinstitute.org/mpg/depict)[20]. To reduce the redundancy of the pathways, we applied affinity propagation clustering implemented in the apcluster package v.1.4.8 in R [28,29]. Using this algorithm, we obtained distinct clusters that contained ‘exemplar’ pathway and member pathways. We extracted 48 exemplar tissues and 1,157 gene sets for enrichment analysis and selected independent AF-associated variants with $\log_{10}$ BF > 5

as applied in a previous study[30]. The significance level was set at $P = 1 \times 10^{-3}$ (=0.05/48) for tissue enrichment analysis and $P = 4.3 \times 10^{-5}$ (=0.05/1,157) for gene set enrichment analysis.

References

1. Lip, G.Y. *et al.* Sex-related differences in presentation, treatment, and outcome of patients with atrial fibrillation in Europe: a report from the Euro Observational Research Programme Pilot survey on Atrial Fibrillation. *Europace* **17**, 24-31 (2015).
2. Schnabel, R.B. *et al.* Gender differences in clinical presentation and 1-year outcomes in atrial fibrillation. *Heart* **103**, 1024-1030 (2017).
3. Ball, J., Carrington, M.J., Wood, K.A., Stewart, S. & Investigators, S. Women versus men with chronic atrial fibrillation: insights from the Standard versus Atrial Fibrillation spEcific managemenT studY (SAFETY). *PLoS One* **8**, e65795 (2013).
4. Benjamin, E.J. *et al.* Impact of atrial fibrillation on the risk of death: the Framingham Heart Study. *Circulation* **98**, 946-52 (1998).
5. Scheuermeyer, F.X. *et al.* There Are Sex Differences in the Demographics and Risk Profiles of Emergency Department (ED) Patients With Atrial Fibrillation and Flutter, but no Apparent Differences in ED Management or Outcomes. *Acad Emerg Med* **22**, 1067-75 (2015).
6. Yang, J. *et al.* Common SNPs explain a large proportion of the heritability for human height. *Nat Genet* **42**, 565-9 (2010).
7. Bulik-Sullivan, B.K. *et al.* LD Score regression distinguishes confounding from polygenicity in genome-wide association studies. *Nat Genet* **47**, 291-5 (2015).
8. Conforto, T.L., Zhang, Y., Sherman, J. & Waxman, D.J. Impact of CUX2 on the female mouse liver transcriptome: activation of female-biased genes and repression of male-biased genes. *Mol Cell Biol* **32**, 4611-27 (2012).
9. Ling, G., Sugathan, A., Mazor, T., Fraenkel, E. & Waxman, D.J. Unbiased, genome-wide in vivo mapping of transcriptional regulatory elements reveals sex differences in chromatin structure associated with sex-specific liver gene expression. *Mol Cell Biol* **30**, 5531-44 (2010).
10. Lin, H. *et al.* Methylome-wide Association Study of Atrial Fibrillation in Framingham Heart Study. *Sci Rep* **7**, 40377 (2017).
11. Battle, A. *et al.* Genetic effects on gene expression across human tissues. *Nature* **550**, 204-

213 (2017).

12. Gudbjartsson, D.F. *et al.* A sequence variant in ZFHX3 on 16q22 associates with atrial fibrillation and ischemic stroke. *Nat Genet* **41**, 876-8 (2009).

13. Zaw, K.T.T. *et al.* Association of ZFHX3 gene variation with atrial fibrillation, cerebral infarction, and lung thromboembolism: An autopsy study. *J Cardiol* **70**, 180-184 (2017).

14. Amendt, B.A., Sutherland, L.B., Semina, E.V. & Russo, A.F. The molecular basis of Rieger syndrome. Analysis of Pitx2 homeodomain protein activities. *J Biol Chem* **273**, 20066-72 (1998).

15. Oki, S. *et al.* ChIP-Atlas: a data-mining suite powered by full integration of public ChIP-seq data. *EMBO Rep* **19**(2018).

16. Ramji, D.P. & Foka, P. CCAAT/enhancer-binding proteins: structure, function and regulation. *Biochem J* **365**, 561-75 (2002).

17. Akiyama, M. *et al.* Genome-wide association study identifies 112 new loci for body mass index in the Japanese population. *Nat Genet* **49**, 1458-1467 (2017).

18. Kanai, M. *et al.* Genetic analysis of quantitative traits in the Japanese population links cell types to complex human diseases. *Nat Genet* **50**, 390-400 (2018).

19. Akiyama, M. *et al.* Characterizing rare and low-frequency height-associated variants in the Japanese population. *Nat Commun* **10**, 4393 (2019).

20. Pers, T.H. *et al.* Biological interpretation of genome-wide association studies using predicted gene functions. *Nat Commun* **6**, 5890 (2015).

21. Nielsen, J.B. *et al.* Biobank-driven genomic discovery yields new insight into atrial fibrillation biology. *Nat Genet* **50**, 1234-1239 (2018).

22. Sharma, M. *et al.* hZimp10 is an androgen receptor co-activator and forms a complex with SUMO-1 at replication foci. *EMBO J* **22**, 6101-14 (2003).

23. Rodriguez-Magadán, H., Merino, E., Schnabel, D., Ramírez, L. & Lomelí, H. Spatial and temporal expression of Zimp7 and Zimp10 PIAS-like proteins in the developing mouse embryo. *Gene Expr Patterns* **8**, 206-13 (2008).

24. Henderson, P., van Limbergen, J.E., Wilson, D.C., Satsangi, J. & Russell, R.K. Genetics of childhood-onset inflammatory bowel disease. *Inflamm Bowel Dis* **17**, 346-61 (2011).

25. Beliakoff, J. *et al.* The PIAS-like protein Zimp10 is essential for embryonic viability and proper vascular development. *Mol Cell Biol* **28**, 282-92 (2008).

26. Giambartolomei, C. *et al.* Bayesian test for colocalisation between pairs of genetic association studies using summary statistics. *PLoS Genet* **10**, e1004383 (2014).

27. Li, Y.I., Wong, G., Humphrey, J. & Raj, T. Prioritizing Parkinson's disease genes using population-scale transcriptomic data. *Nat Commun* **10**, 994 (2019).

28. Frey, B.J. & Dueck, D. Clustering by passing messages between data points. *Science* **315**, 972-6 (2007).

29. Marouli, E. *et al.* Rare and low-frequency coding variants alter human adult height. *Nature* **542**, 186-190 (2017).

30. Malik, R. *et al.* Multiancestry genome-wide association study of 520,000 subjects identifies 32 loci associated with stroke and stroke subtypes. *Nat Genet* **50**, 524-537 (2018).

List of Consortium Members

BioBank Japan Project Consortium

Koichi Matsuda. Laboratory of Genome Technology, Human Genome Center, Institute of Medical Science, The University of Tokyo, Tokyo, Japan. Laboratory of Clinical Genome Sequencing, Graduate School of Frontier Sciences, The University of Tokyo, Tokyo, Japan. kmatsuda@edu.k.u-tokyo.ac.jp

Yuji Yamanashi. Division of Genetics, The Institute of Medical Science, The University of Tokyo, Tokyo, Japan. yyamanas@ims.u-tokyo.ac.jp

Yoichi Furukawa. Division of Clinical Genome Research, Institute of Medical Science, The University of Tokyo, Tokyo, Japan. furukawa@ims.u-tokyo.ac.jp

Takayuki Morisaki. Division of Molecular Pathology Genomic Medicine and Disease Prevention The Institute of Medical IMSUT Hospital, Department of Internal Medicine Project Division of Science The University of Tokyo, Tokyo, Japan. morisaki@ims.u-tokyo.ac.jp

Yoshinori Murakami. Department of Cancer Biology, Institute of Medical Science, The University of Tokyo, Tokyo, Japan. ymurakam@ims.u-tokyo.ac.jp

Yoichiro Kamatani. Laboratory of Complex Trait Genomics, Graduate School of Frontier Sciences, The University of Tokyo. Laboratory of Clinical Genome Sequencing, Graduate School of Frontier Sciences, The University of Tokyo, Tokyo, Japan. kamatani.yoichiro@edu.k.u-tokyo.ac.jp

Kaori Muto. Department of Public Policy, Institute of Medical Science, The University of Tokyo, Tokyo, Japan. krmt@ims.u-tokyo.ac.jp

Akiko Nagai. Department of Public Policy, Institute of Medical Science, The University of Tokyo, Tokyo, Japan. akikongi@ims.u-tokyo.ac.jp

Wataru Obara. Department of Urology, Iwate Medical University, Iwate, Japan. watao@iwate-med.ac.jp

Ken Yamaji. Department of Internal Medicine and Rheumatology, Juntendo University Graduate School of Medicine, Tokyo, Japan. k.yamaji@juntendo.ac.jp

Kazuhisa Takahashi. Department of Respiratory Medicine, Juntendo University Graduate School of Medicine, Tokyo, Japan. kztakaha@juntendo.ac.jp

Satoshi Asai. Division of Pharmacology, Department of Biomedical Science, Nihon University School of Medicine, Tokyo, Japan. Division of Genomic Epidemiology and Clinical Trials, Clinical Trials Research Center, Nihon University. School of Medicine, Tokyo, Japan. asai.satoshi@nihon-u.ac.jp

Yasuo Takahashi. Division of Genomic Epidemiology and Clinical Trials, Clinical Trials Research Center, Nihon University School of Medicine, Tokyo, Japan. takahashi.yasuo@nihon-u.ac.jp

Takao Suzuki. Tokushukai Group, Tokyo, Japan. takao.suzuki@tokushukai.jp

Nobuaki Sinozaki. Tokushukai Group, Tokyo, Japan. nobuaki.shinozaki@tokushukai.jp

Hiroki Yamaguchi. Departmentof Hematology, Nippon Medical School, Tokyo, Japan. y-hiroki@fd6.so-net.ne.jp

Shiro Minami. Department of Bioregulation, Nippon Medical School, Kawasaki, Japan. shirom@nms.ac.jp

Shigeo Murayama. Tokyo Metropolitan Geriatric Hospital and Institute of Gerontology, Tokyo, Japan. smurayam@bbarjp.net

Kozo Yoshimori. Fukujuji Hospital, Japan Anti-Tuberculosis Association, Tokyo, Japan. yoshimorik@fukujuji.org

Satoshi Nagayama. The Cancer Institute Hospital of the Japanese Foundation for Cancer Research, Tokyo, Japan. snagayama2009@hotmail.co.jp

Daisuke Obata. Center for Clinical Research and Advanced Medicine, Shiga University of Medical Science, Shiga, Japan. dobata@belle.shiga-med.ac.jp

Masahiko Higashiyama. Department of General Thoracic Surgery, Osaka International Cancer Institute, Osaka, Japan. higashiyama-m@higashiosaka-hosp.jp

Akihide Masumoto. IIZUKA HOSPITAL, Fukuoka,Japan. amasumotoh1@aih-net.com

Yukihiro Koretsune. National Hospital Organization Osaka National Hospital, Osaka, Japan. koretune318@hotmail.co.jp

Supplementary Figures

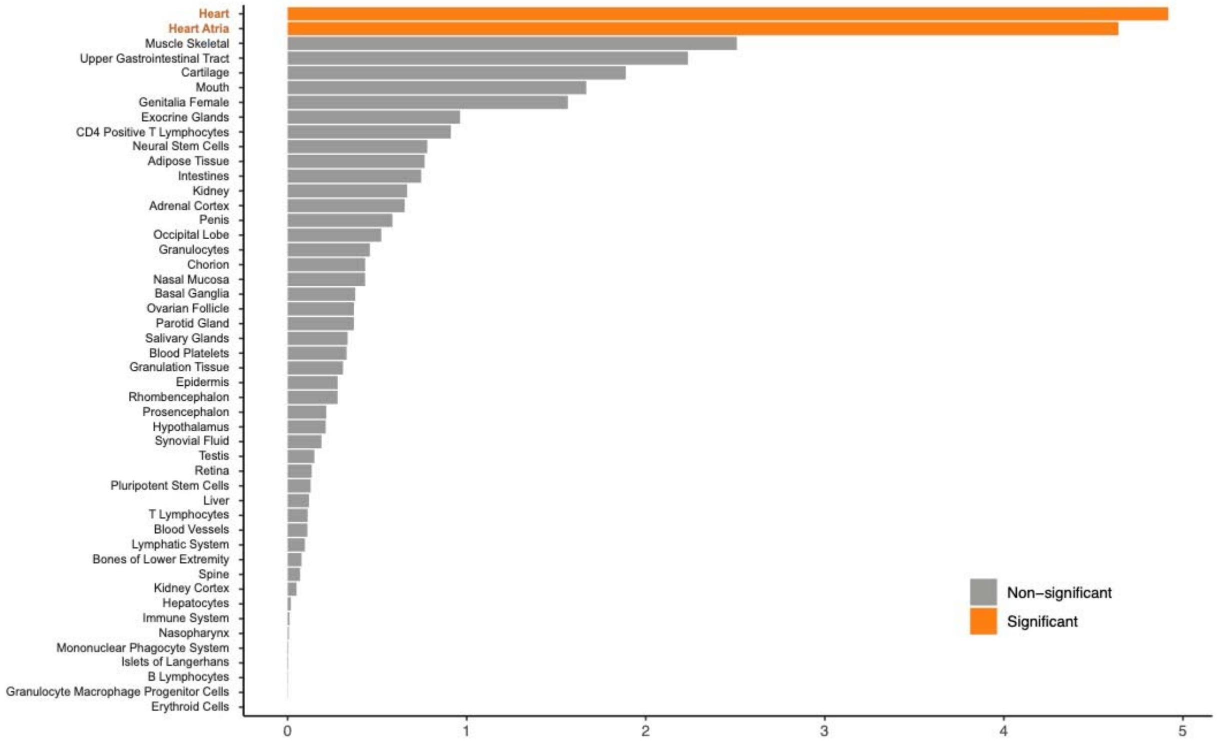

Supplementary Fig. 1 | Tissue enrichment analysis.

The results of tissue enrichment analysis using DEPICT software. AF-associated variants with $\log_{10}$ BF > 5 were selected (n = 16,181). The *x-axis* indicates the $-\log_{10} P$ value. Forty-eight exemplar tissues (Methods) were tested, and the significance level was set at $P = 1 \times 10^{-3}$ (0.05/48) accounting for multiple testing of tested tissues. BF, Bayes factor.

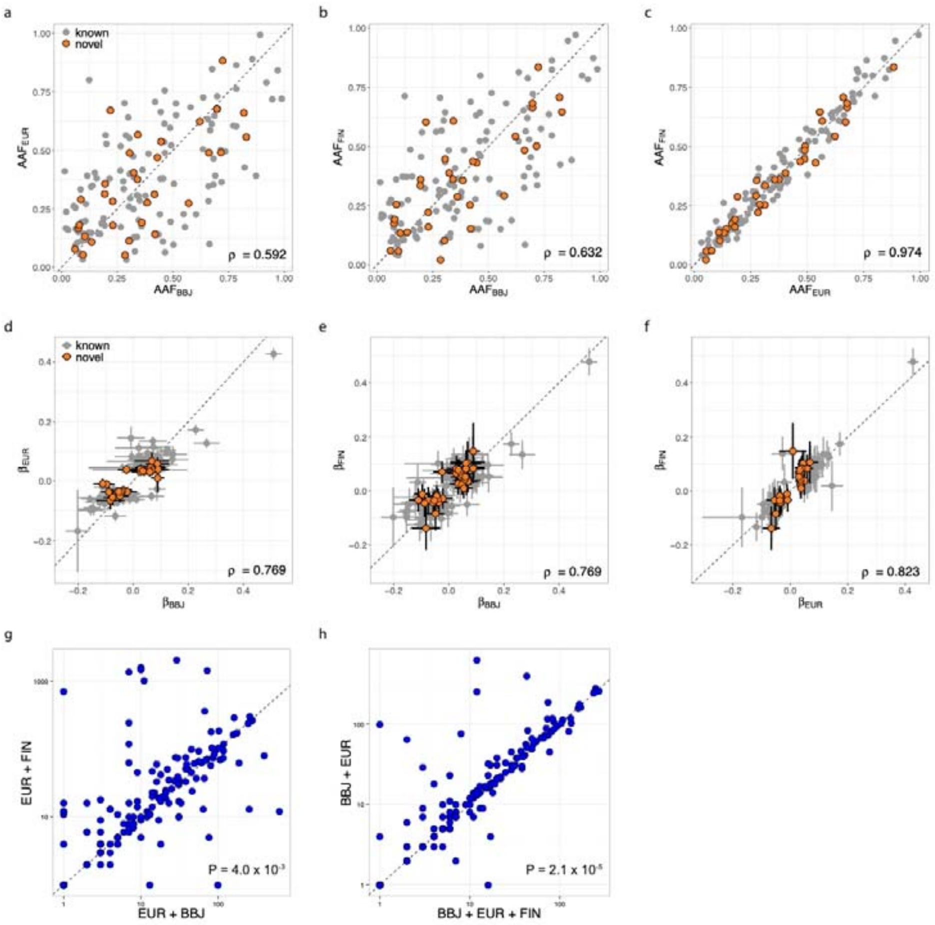

Supplementary Fig. 2 | Comparison of allele frequencies and allelic effects among ancestries and fine mapping derived from credible set analyses.

Data on the results from each GWAS are shown: *n* = 150,272 (9,826 cases and 140,446 controls) for BBJ; *n* = 1,030,836 (60,620 cases and 970,216 controls) for EUR; *n* = 63,622 (7,244 cases and 56,378 controls) for FIN; *n* = 1,181,108 (70,446 cases and 1,110,662 controls) for BBJ+EUR; *n* = 1,094,458 (67,864 cases and 1,026,594 controls) for EUR+FIN; *n* = 1,244,730 (77,690 cases and 1,167,040 controls) for BBJ+EUR+FIN. **a-c**, Comparisons of alternate allele frequencies of the 150 lead variants identified in the cross-ancestry meta-analysis (**a**, BBJ versus EUR; **b**, BBJ versus FIN; **c**, EUR versus FIN). **d-f**, Comparison of estimated effect sizes of 150 lead variants. Effect sizes and confidence intervals were calculated using a logistic regression model. Each dot represents an estimated effect size (β) with an error bar indicating the 95% CI of the estimate (**d**, BBJ versus EUR; **e**, BBJ versus FIN; **f**, EUR versus FIN). Grey points indicate previously reported loci, and orange

points indicate newly identified loci in this study. ρ indicates Spearman's correlation coefficient. **g**, **h**, Comparisons of the number of variants included in the 99% credible sets derived from each combination of the meta-analysis using paired Wilcoxon rank-sum test (**g**, EUR + BBJ versus EUR + FIN; **h**, BBJ + EUR + FIN versus BBJ + EUR). Both *the x* and *y* axes represent the log-transformed number of variants in the 99% credible sets. BBJ, BioBank Japan; EUR, European population; FIN, FinnGen data release 2; CI, confidence interval.

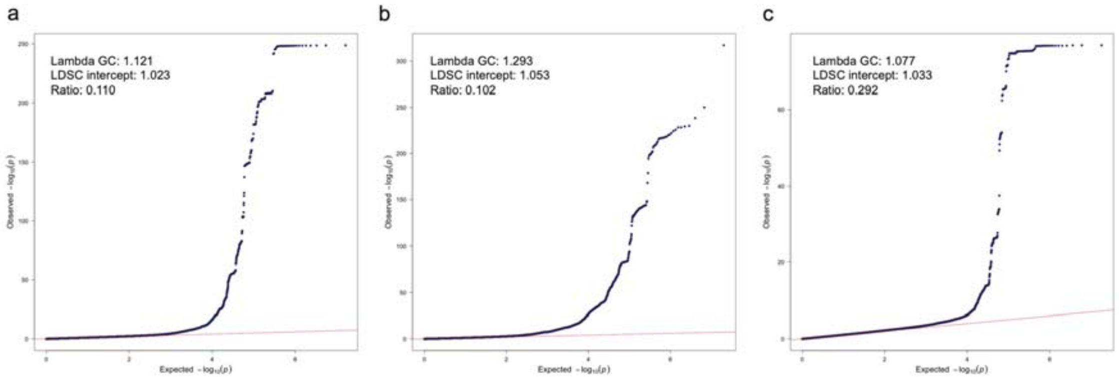

Supplementary Fig. 3 | Quantile-quantile plot for GWAS of three studies.

a, the Japanese GWAS. **b**, GWAS of a meta-analysis in European population (EUR). **c**, GWAS in data from FinnGen project (FIN). GWAS, genome-wide association study. In **a**, **b**, and **c**, two-sided *P* values were calculated using a logistic regression model.

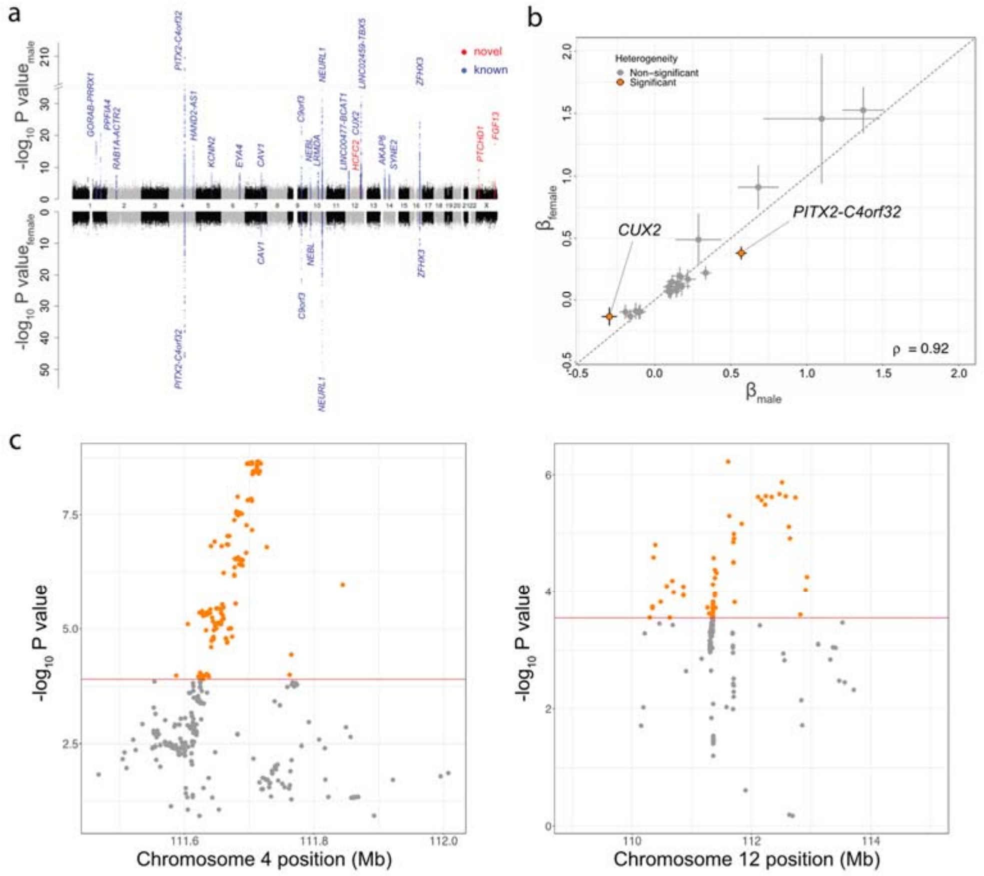

Supplementary Fig. 4 | Sex-stratified GWAS.

Number of samples in sex-stratified GWAS was as follows: n = 81,050 (6,825 cases and 74,225 controls) for male GWAS; n = 69,222 (3,001 cases and 66,221 controls) for female GWAS. **a**, Miami plot for sex-stratified GWAS. Upper panel for male GWAS and lower panel for female GWAS. Association signals that reached a genome-wide significance level ($P < 5.0 \times 10^{-8}$) are shown in blue if previously reported loci and in red if novel loci. Two-sided P values were calculated using a logistic regression model. **b**, Comparison of estimated effect sizes of the lead variants identified in sex-stratified GWAS (n = 27 SNPs). Each dot represents an estimated effect size (β) with an error bar indicating the 95% CI of the estimate. The lead variants with significant heterogeneity ($P_{het} < 0.0001$) are highlighted in orange, and ρ indicates Spearman's correlation coefficient (two-sided P value = 7.4 × 10^{-7}). GWAS, genome-wide association study. **c**, The results of the omnibus test in the *PITX2-C4orf32* (left) and *CUX2* loci (right), respectively. Chi-square statistics were calculated from the omnibus test using regression models with and without an interaction term of SNP × sex. The y axis

represents $-\log_{10}$ *P* values obtained from the omnibus test for AF-associated variants with heterogeneity between sexes, showing against the genomic position of chromosomes 4 (left) and 12 (right) on the *x* axis. The red line indicates a significant threshold adjusted for the number of variants tested (*P* = 0.05/399 for the *PITX2-C4orf32* locus and *P* = 0.05/176 for the *CUX2* locus).

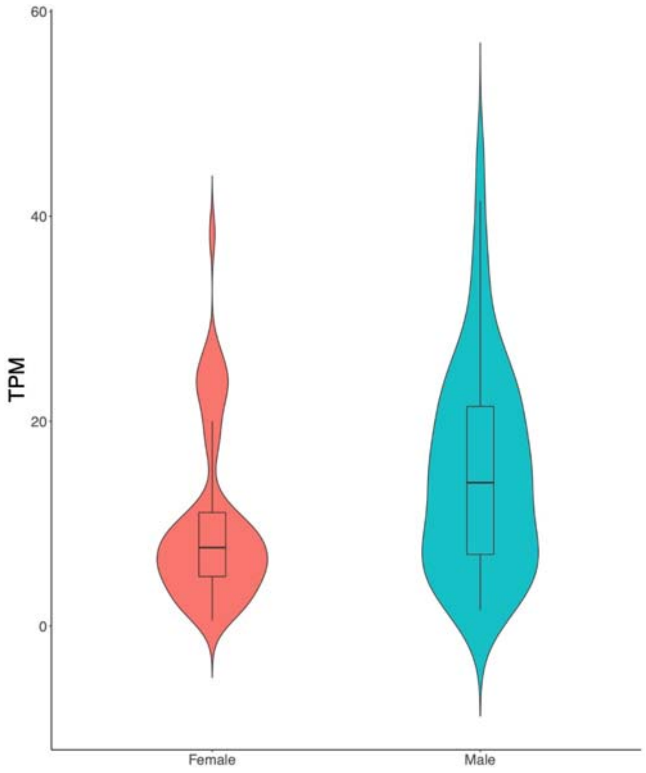

Supplementary Fig. 5 | *CUX2* gene expression in liver.

Violin plots showing the distribution of *CUX2* gene expression in the liver based on GTEx data (n_{female}=55 and n_{male}=120). Box plot represents the median, the bounds represent the first and third quartile, and the whiskers reach to 1.5 times the interquartile range.

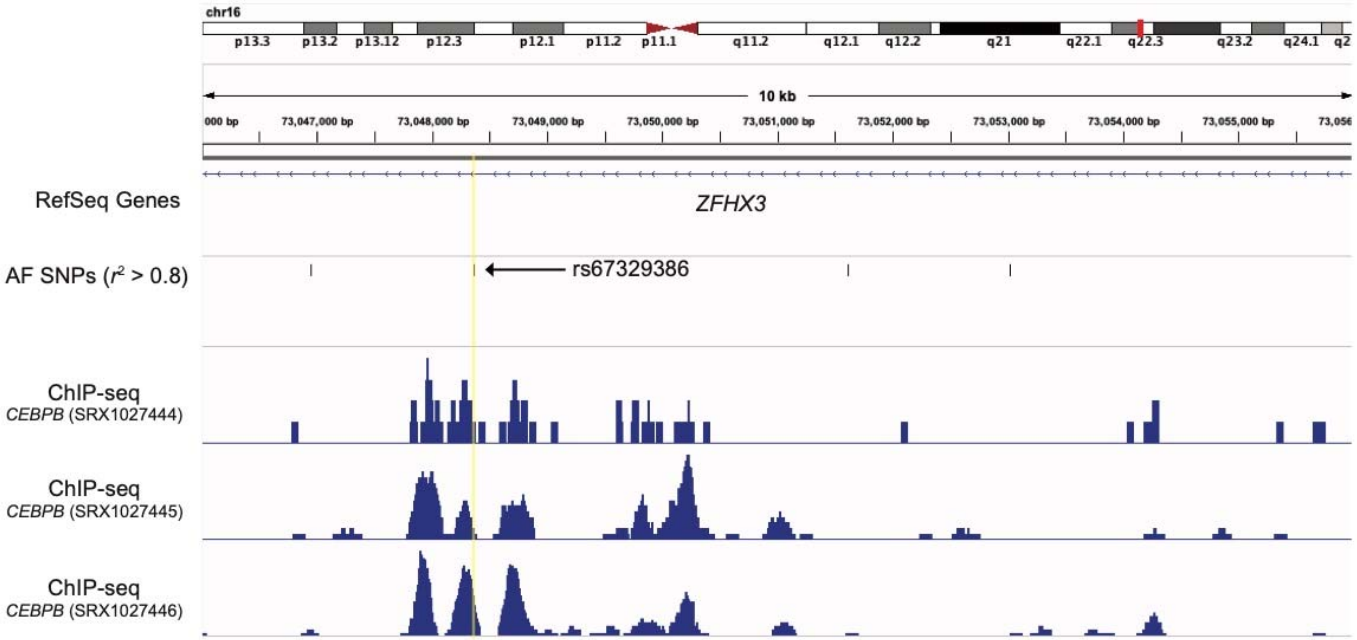

Supplementary Fig. 6 | rs67329386 and transcription factor.

ZFHX3 locus showing rs67329386 (highlighted in yellow) and proxies with r^2 > 0.8 in European samples in 1KG along with ChIP-seq track of CEBPB experiments in mesenchymal stem cells.

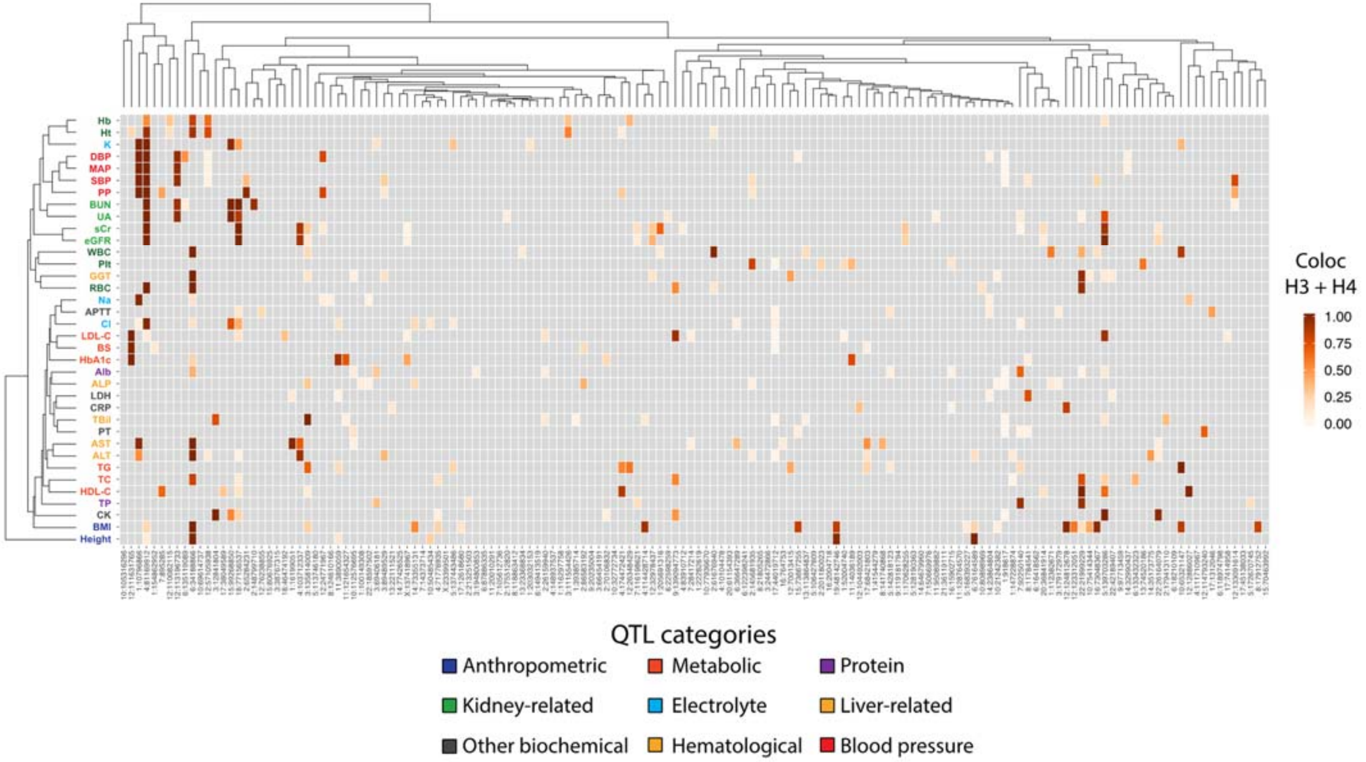

Supplementary Fig. 7 | Colocalization of quantitative traits with AF-associated signals.

Heat-map representation of the approximate Bayes factor posterior probability of AF and quantitative traits to share a common causal variant at 150 AF-associated loci (coloc H3 + H4 represented on a white-red color scale and H4/H3 < 2 in grey). The rows show 36 quantitative traits highlighted in each color of the 9 categories, and the columns show the chromosome and physical position of the lead variant in AF-associated loci. AF, atrial fibrillation; QTL, quantitative trait loci.
