# Supplementary material for: Cross-ancestry genome-wide analysis of atrial fibrillation unveils disease biology and enables cardioembolic risk prediction
Source: Nat Genet. 2023 Jan 19;55(2):187–97. doi: 10.1038/s41588-022-01284-9 (PMC9925380; doi:10.1038/s41588-022-01284-9)
Supplement: Supplementary file 2 — Reporting Summary [file 41588_2022_1284_MOESM2_ESM.pdf]

nature portfolio

Corresponding author(s): Kaoru Ito
Last updated by author(s): Dec 9, 2022

Reporting Summary

Nature Portfolio wishes to improve the reproducibility of the work that we publish. This form provides structure for consistency and transparency in reporting. For further information on Nature Portfolio policies, see our Editorial Policies and the Editorial Policy Checklist.

Statistics

For all statistical analyses, confirm that the following items are present in the figure legend, table legend, main text, or Methods section.

n/a	Confirmed	
☐	☒	The exact sample size (*n*) for each experimental group/condition, given as a discrete number and unit of measurement
☐	☒	A statement on whether measurements were taken from distinct samples or whether the same sample was measured repeatedly
☐	☒	The statistical test(s) used AND whether they are one- or two-sided<br>*Only common tests should be described solely by name; describe more complex techniques in the Methods section.*
☐	☒	A description of all covariates tested
☐	☒	A description of any assumptions or corrections, such as tests of normality and adjustment for multiple comparisons
☐	☒	A full description of the statistical parameters including central tendency (e.g. means) or other basic estimates (e.g. regression coefficient) AND variation (e.g. standard deviation) or associated estimates of uncertainty (e.g. confidence intervals)
☐	☒	For null hypothesis testing, the test statistic (e.g. *F*, *t*, *r*) with confidence intervals, effect sizes, degrees of freedom and *P* value noted<br>*Give P values as exact values whenever suitable.*
☒	☐	For Bayesian analysis, information on the choice of priors and Markov chain Monte Carlo settings
☒	☐	For hierarchical and complex designs, identification of the appropriate level for tests and full reporting of outcomes
☐	☒	Estimates of effect sizes (e.g. Cohen's *d*, Pearson's *r*), indicating how they were calculated

Our web collection on statistics for biologists contains articles on many of the points above.

Software and code

Policy information about availability of computer code

Data collection	No software was used for data collection.
Data analysis	We used open source softwares for the analysis, as listed below.<br><br>bgzip/tabix (0.2.6), http://www.htslib.org/doc/tabix.html;<br>Minimac3 (2.0.1), https://genome.sph.umich.edu/wiki/Minimac3;<br>Minimac4 (1.0.0), https://genome.sph.umich.edu/wiki/Minimac4;<br>Eagle (2.4.1), https://data.broadinstitute.org/alkesgroup/Eagle;<br>SHAPEIT2 (r837), https://mathgen.stats.ox.ac.uk/genetics_software/shapeit/shapeit.html;<br>ANNOVAR (2017Jun01), http://annovar.openbioinformatics.org;<br>Open Targets, https://www.opentargets.org;<br>PLINK (2.0), https://www.cog-genomics.org/plink;<br>LDSC (1.0.0), https://github.com/bulik/ldsc;<br>GCTA-GREML, http://cnsgenomics.com/software/gcta/#BivariateGREMLanalysis;<br>Genetic Association Study Power Calculator, https://csg.sph.umich.edu/abecasis/gas_power_calculator;<br>MANTRA (2.0), provided by the author;<br>METASOFT (2.0), http://genetics.cs.ucla.edu/meta;<br>METAL (2011-03-25), https://genome.sph.umich.edu/wiki/METAL;<br>bedtools (2.25.0), https://bedtools.readthedocs.io/en/latest;<br>LocusZoom (1.4), http://locuszoom.sph.umich.edu;<br>R (3.5.1), https://www.r-project.org;<br>python (4.0.3), https://www.python.org;<br>DEPICT (1, release 194), https://data.broadinstitute.org/mpg/depict;

SpliceAI (v.1.3.1), https://github.com/Illumina/SpliceAI;
Popcorn software (1.0), https://github.com/brielin/Popcorn;
MetaXcan (0.3.5), https://github.com/hakyimlab/MetaXcan/wiki;
FUMA (v1.3.7), https://fuma.ctglab.nl;
LDpred2, https://privefl.github.io/bigsnpr/articles/LDpred2.html;
TwoSampleMR, https://github.com/MRCIEU/TwoSampleMR;
MRPRESSO (1.0), https://github.com/rondolab/MR-PRESSO;
PhenoScanner V2, http://www.phenoscanner.medschl.cam.ac.uk;
GraphPad Prism 7.04, https://www.graphpad.com/scientific-software/prism;
coxph in the R survival package v2.44

Further details are described in the Method section.

For manuscripts utilizing custom algorithms or software that are central to the research but not yet described in published literature, software must be made available to editors and reviewers. We strongly encourage code deposition in a community repository (e.g. GitHub). See the Nature Portfolio guidelines for submitting code & software for further information.

Data

Policy information about availability of data

All manuscripts must include a data availability statement. This statement should provide the following information, where applicable:

- Accession codes, unique identifiers, or web links for publicly available datasets
- A description of any restrictions on data availability
- For clinical datasets or third party data, please ensure that the statement adheres to our policy

We used publicly available data   as listed below.

Summary results of the European AF meta-analysis are publicly available on http://csg.sph.umich.edu/willer/public/afib2018.
Summary results from the FinnGen release 2 data can be accessed through application on https://www.finngen.fi/en/access_results.
Summary statistics of quantitative trait loci in the BioBank Japan are publicly available on http://jenger.riken.jp/en/ (JENGER).
Summary statistics of the UK Biobank are publicly available on http://www.nealelab.is/uk-biobank.

ggnomAD, https://gnomad.broadinstitute.org;
ClinVar, https://www.ncbi.nlm.nih.gov/clinvar;
1000 Genomes Project, http://www.1000genomes.org;
HapMap project, http://hapmap.ncbi.nlm.nih.gov;
GWAS catalog, https://www.ebi.ac.uk/gwas;
dbSNP, https://www.ncbi.nlm.nih.gov/snp;
PubTator, https://www.ncbi.nlm.nih.gov/research/pubtator;
GTEx, https://gtexportal.org/home;
ChIP-Atlas, https://chip-atlas.org;
ENCODE, https://www.encodeproject.org

Further details are described in the Method section.

The summary statistics of the Japanese GWAS and the cross-ancestry meta-analysis, and the data for the calculation of PRS derived from the current study are publicly available in the National Bioscience Database Center (research ID: hum0014, https://humandbs.biosciencedbc.jp/en/). The phenotype information can be provided by the BioBank Japan project upon a request (https://biobankjp.org/english/index.html).

Field-specific reporting

Please select the one below that is the best fit for your research. If you are not sure, read the appropriate sections before making your selection.

☒ Life sciences   ☐ Behavioural & social sciences   ☐ Ecological, evolutionary & environmental sciences

For a reference copy of the document with all sections, see nature.com/documents/nr-reporting-summary-flat.pdf

Life sciences study design

All studies must disclose on these points even when the disclosure is negative.

Sample size	Because we aimed to create the largest sample size in the Japanese population in order to gain the statistical power, we included as many case and control individuals as possible in our analysis.
Data exclusions	We excluded individuals according to the standard quality control procedure of GWAS. Further details are described in the Method section.
Replication	Our Japanese GWAS identified 31 genome-wide significant loci, where 26 previously reported loci were replicated. We also performed a replication study for the newly identified five loci using an independent Japanese cohort, the BioBank Japan second cohort (4,602 cases and 44,075 controls), and confirmed that all signals were successfully replicated with nominal associations ($P < 0.05$) in the same effect direction.
Randomization	Randomization is not applicable, because this is a population based case-control analysis.

Blinding	Blinding is not applicable, because this is a population based case-control analysis.

Reporting for specific materials, systems and methods

We require information from authors about some types of materials, experimental systems and methods used in many studies. Here, indicate whether each material, system or method listed is relevant to your study. If you are not sure if a list item applies to your research, read the appropriate section before selecting a response.

Materials & experimental systems

n/a	Involved in the study
☒	☐ Antibodies
☐	☒ Eukaryotic cell lines
☒	☐ Palaeontology and archaeology
☒	☐ Animals and other organisms
☐	☒ Human research participants
☒	☐ Clinical data
☒	☐ Dual use research of concern

Methods

n/a	Involved in the study
☒	☐ ChIP-seq
☒	☐ Flow cytometry
☒	☐ MRI-based neuroimaging

Eukaryotic cell lines

Policy information about cell lines

Cell line source(s)	All cell lines were iPCs from the University of Tokyo (IRB #11044). Peripheral blood mononuclear cells were obtained from healthy volunteers with their consent, and iPS cells were established using episomal plasmid vectors encoding the reprogramming factors.
Authentication	All cell lines were confirmed to be normal by karyotype analysis, and pluripotency was confirmed by RT-PCR and immunostaining of stem cell markers such as SOX2, POU5F1, and NANOG . All cell lines were matched to the original donor by genotype, using STR anaysis.
Mycoplasma contamination	All cell lines were tested with Mycoplasma using MycoAlert Plus Mycoplasma detection kit (Lonza) and were found negative.
Commonly misidentified lines (See ICLAC register)	NA

Human research participants

Policy information about studies involving human research participants

Population characteristics	Population characteristics such as the number of male and female subjects, the mean and standard deviation of the age in the Japanese GWAS are provided in Supplementary Table 11.
Recruitment	The BioBank Japan (BBJ) project<br>The BBJ project (https://biobankjp.org/english.) is a hospital-based biobank. Participants were recruited at 12 medical institutes throughout Japan. Detailed information is described in the following papers: Nagai A. et al. Overview of the BioBank Japan Project: Study design and profile. J Epidemiol 27, S2–S8 (2017), Hirata M. et al. Cross-sectional analysis of BioBank Japan clinical data: A large cohort of 200,000 patients with 47 common diseases. J Epidemiol 27, S9–S21 (2017).
Ethics oversight	Our study was approved by the appropriate Institutional Review Board at each facility and the informed consent was obtained from all participants in each study. We obtained approval from ethics committees of (1) RIKEN Center for Integrative Medical Sciences, (2) the Institute of Medical Sciences, The University of Tokyo, (3) Kyoto University, (4) National Cancer Center Japan, (5) Nagoya University, (6) Aichi Cancer Center, (7) Osaka University.

Note that full information on the approval of the study protocol must also be provided in the manuscript.
