# Supplementary material for: Cross-ancestry genome-wide analysis of atrial fibrillation unveils disease biology and enables cardioembolic risk prediction
Source: Nat Genet. 2023 Jan 19;55(2):187–97. doi: 10.1038/s41588-022-01284-9 (PMC9925380; doi:10.1038/s41588-022-01284-9)
Supplement: Supplementary file 5 — Supplementary Datasets 1–4. [file 41588_2022_1284_MOESM5_ESM.pdf]

Supplementary Dataset

1. Novel 5 loci in the Japanese GWAS
2. Previously reported 26 loci in the Japanese GWAS
3. Novel 33 loci in the trans-ancestry meta-GWAS
4. Previously reported 127 loci in the trans-ancestry meta-GWAS

1. Novel 5 loci in the Japanese GWAS

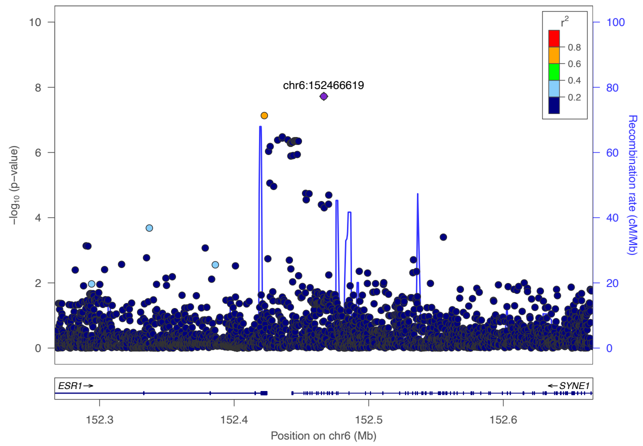

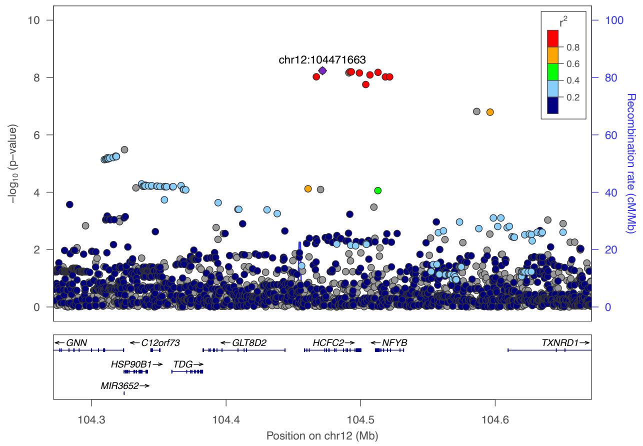

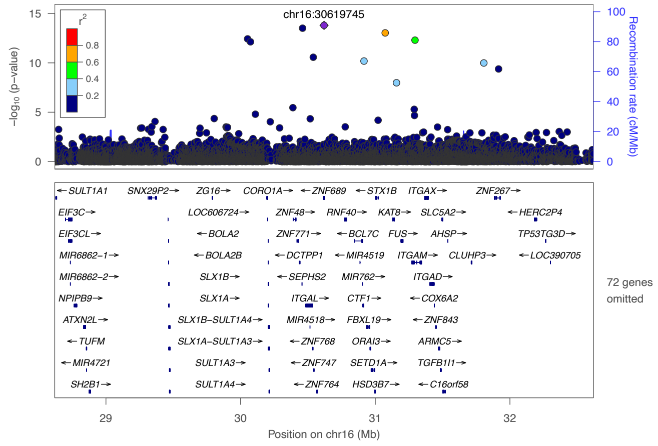

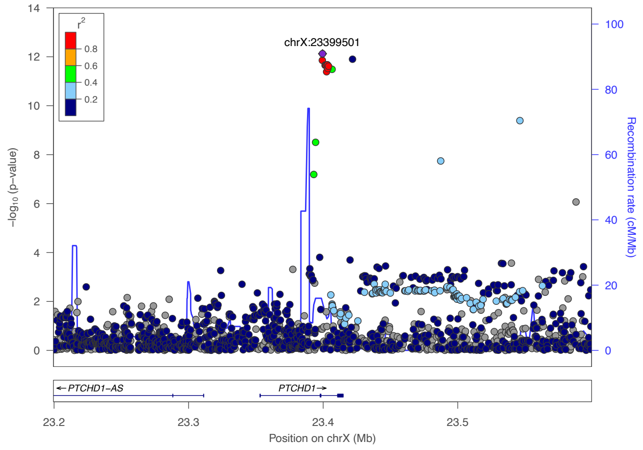

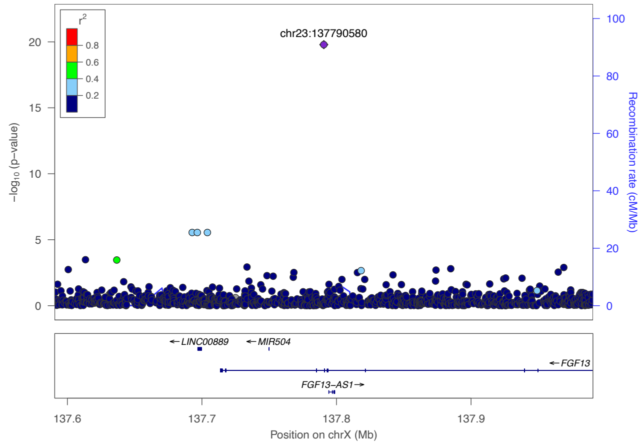

2. Previously reported 26 loci in the Japanese GWAS

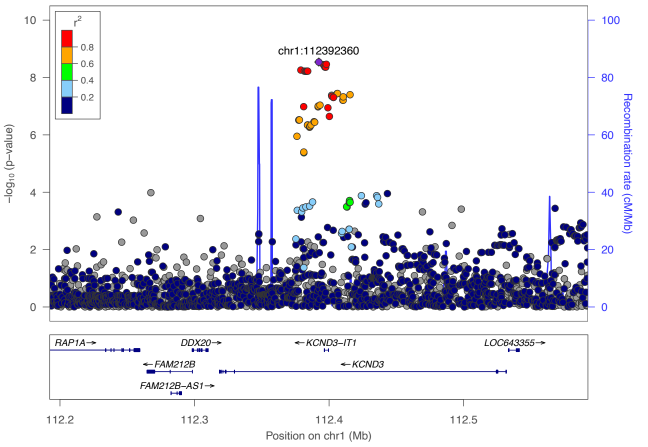

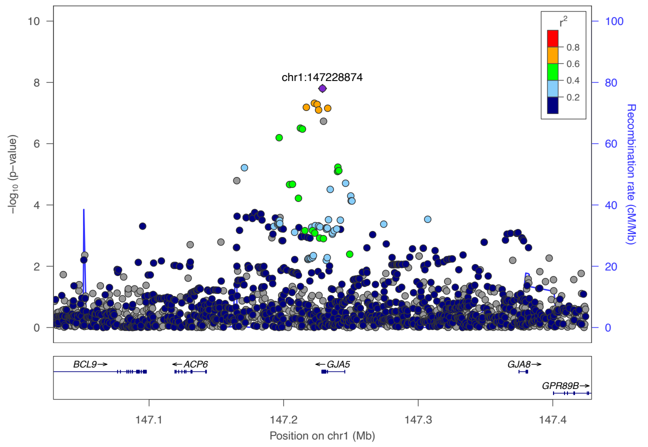

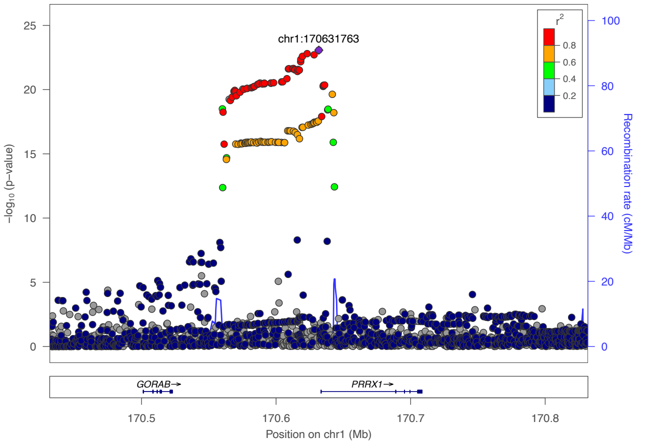

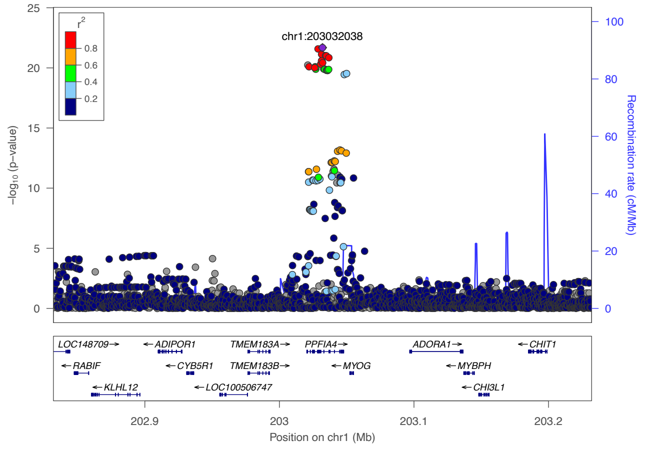

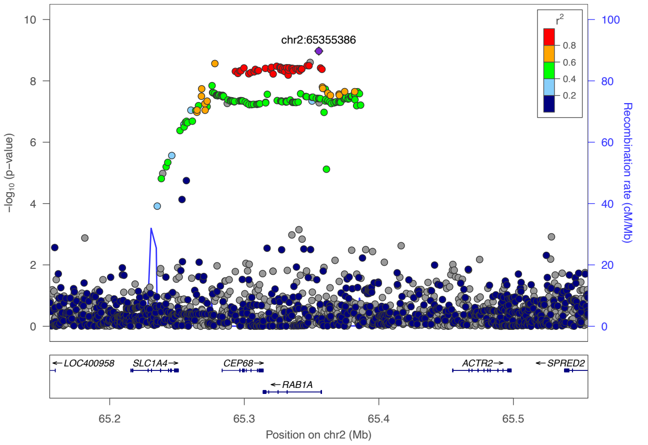

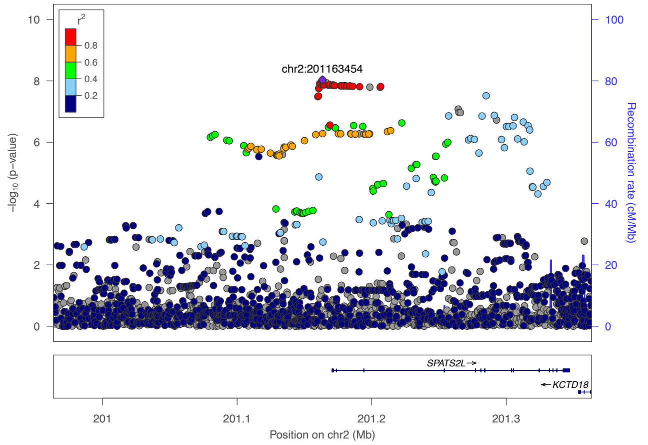

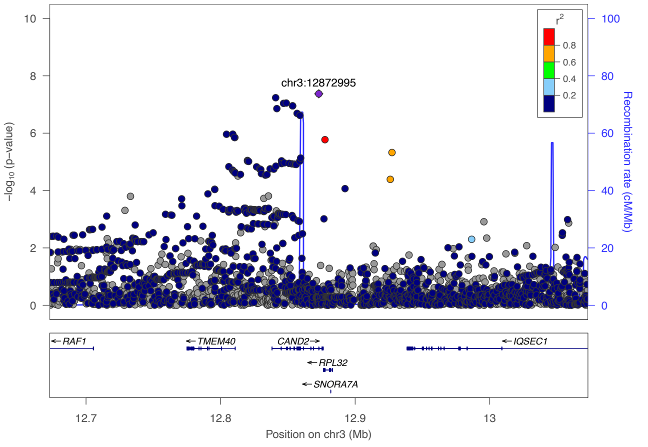

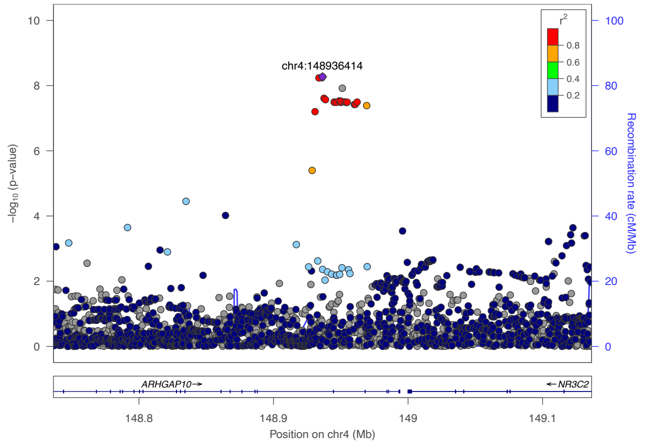

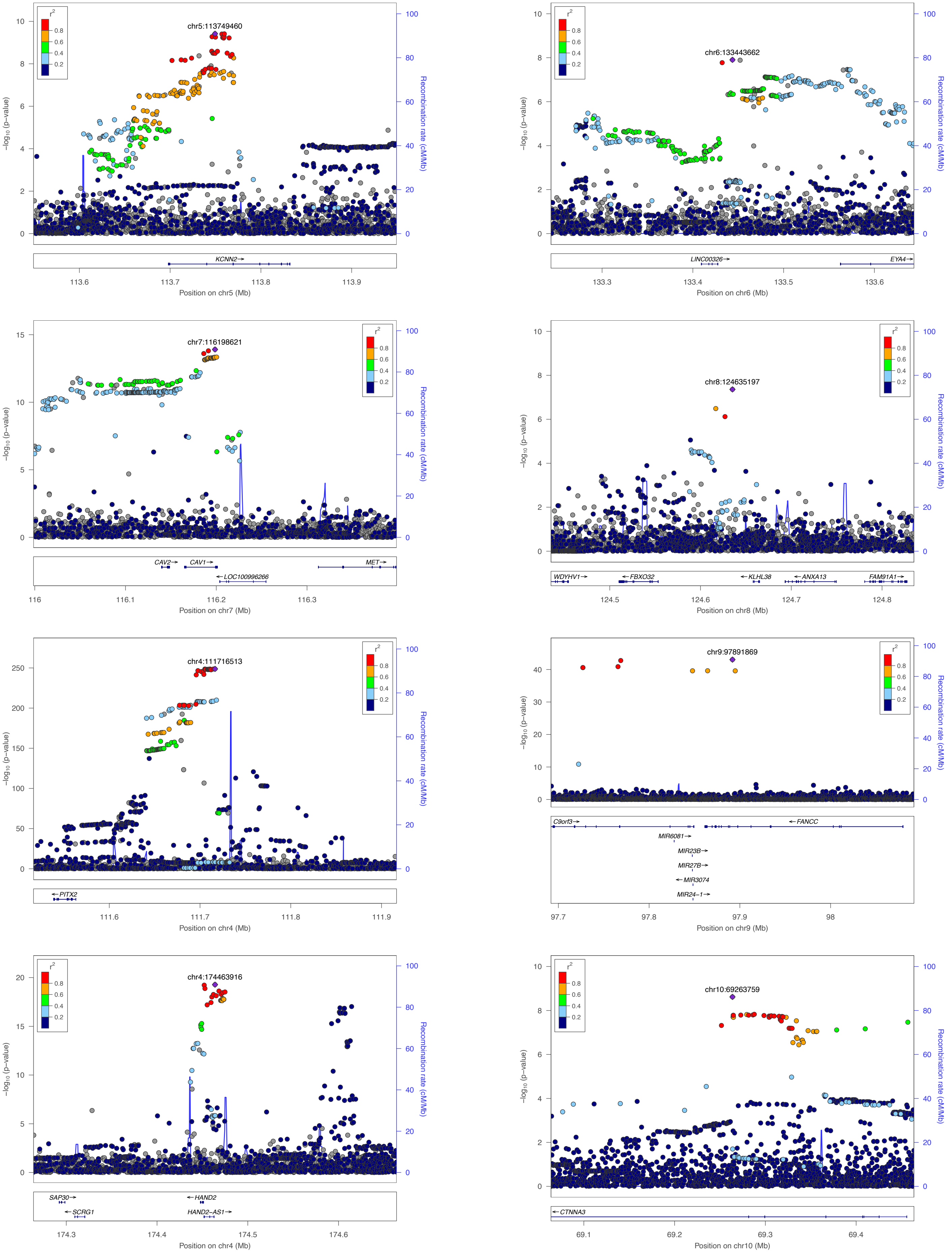

chr5:113749460
KCNN2
Position on chr5 (Mb)
chr6:133443662
LINC00326
EYA4
Position on chr6 (Mb)
chr7:116198621
CAV2
CAV1
MET
LOC100996266
Position on chr7 (Mb)
chr8:124635197
WDYHV1
FBXO32
KLHL38
ANXA13
FAM91A1
Position on chr8 (Mb)
chr4:111716513
PITX2
Position on chr4 (Mb)
chr9:97891869
C9orf3
FANCC
MIR6081
MIR23B
MIR27B
MIR3074
MIR24−1
Position on chr9 (Mb)
chr4:174463916
SAP30
SCRG1
HAND2
HAND2−AS1
Position on chr4 (Mb)
chr10:69263759
CTNNA3
Position on chr10 (Mb)
−log10 (p−value)
Recombination rate (cM/Mb)
r2

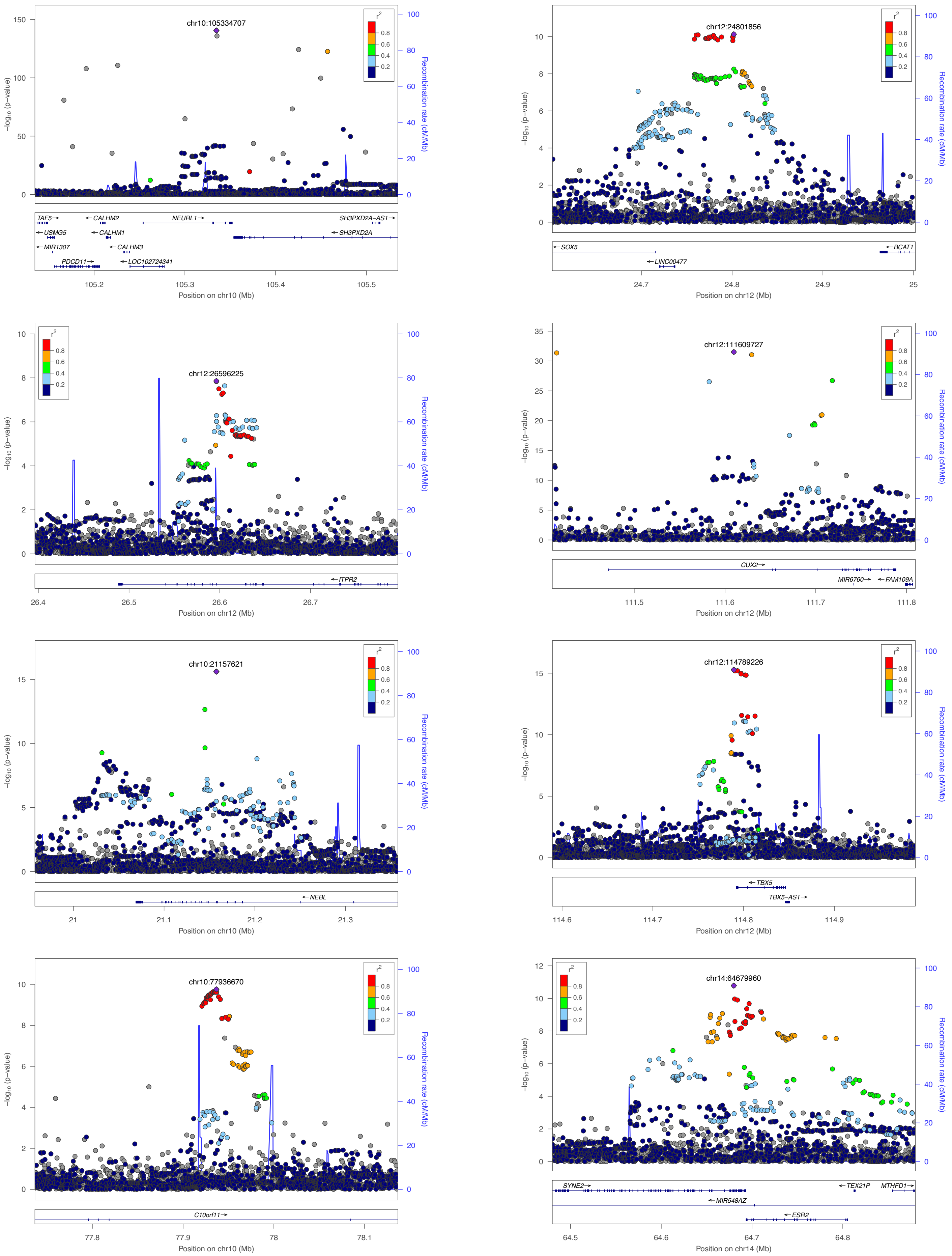

chr10:105334707
−log10 (p−value)
Recombination rate (cM/Mb)
TAF5
USMG5
MIR1307
PDCD11
CALHM2
CALHM1
CALHM3
LOC102724341
NEURL1
SH3PXD2A−AS1
SH3PXD2A
Position on chr10 (Mb)
chr12:24801856
SOX5
LINC00477
BCAT1
Position on chr12 (Mb)
chr12:26596225
ITPR2
Position on chr12 (Mb)
chr12:111609727
CUX2
MIR6760
FAM109A
Position on chr12 (Mb)
chr10:21157621
NEBL
Position on chr10 (Mb)
chr12:114789226
TBX5
TBX5−AS1
Position on chr12 (Mb)
chr10:77936670
C10orf11
Position on chr10 (Mb)
chr14:64679960
SYNE2
MIR548AZ
ESR2
TEX21P
MTHFD1
Position on chr14 (Mb)

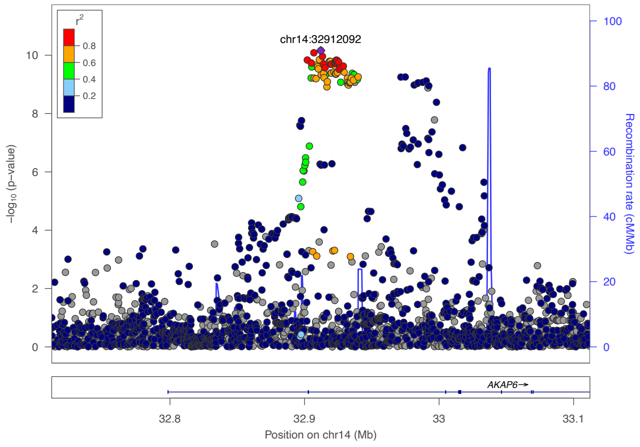
chr14:32912092
r2
0.8
0.6
0.4
0.2
−log10 (p−value)
Recombination rate (cM/Mb)
AKAP6→
32.8
32.9
33
33.1
Position on chr14 (Mb)

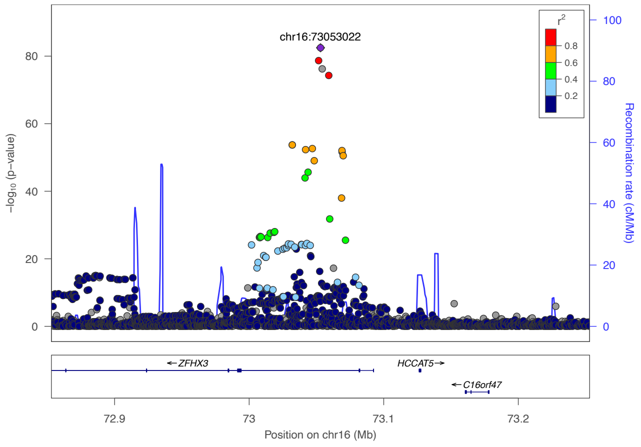
chr16:73053022
r2
0.8
0.6
0.4
0.2
−log10 (p−value)
Recombination rate (cM/Mb)
←ZFHX3
HCCAT5→
←C16orf47
72.9
73
73.1
73.2
Position on chr16 (Mb)

3. Novel 33 loci in the trans-ancestry meta-GWAS

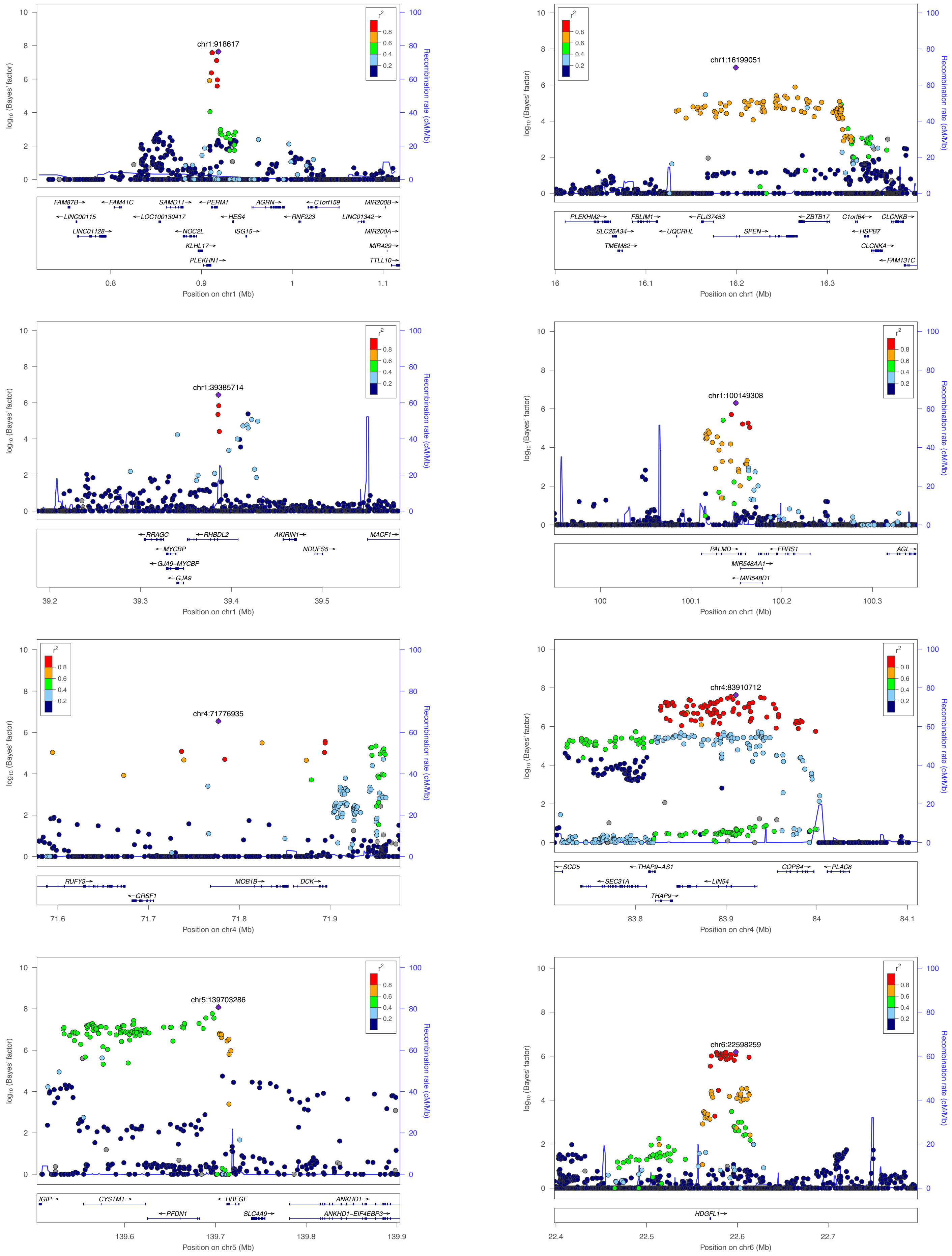

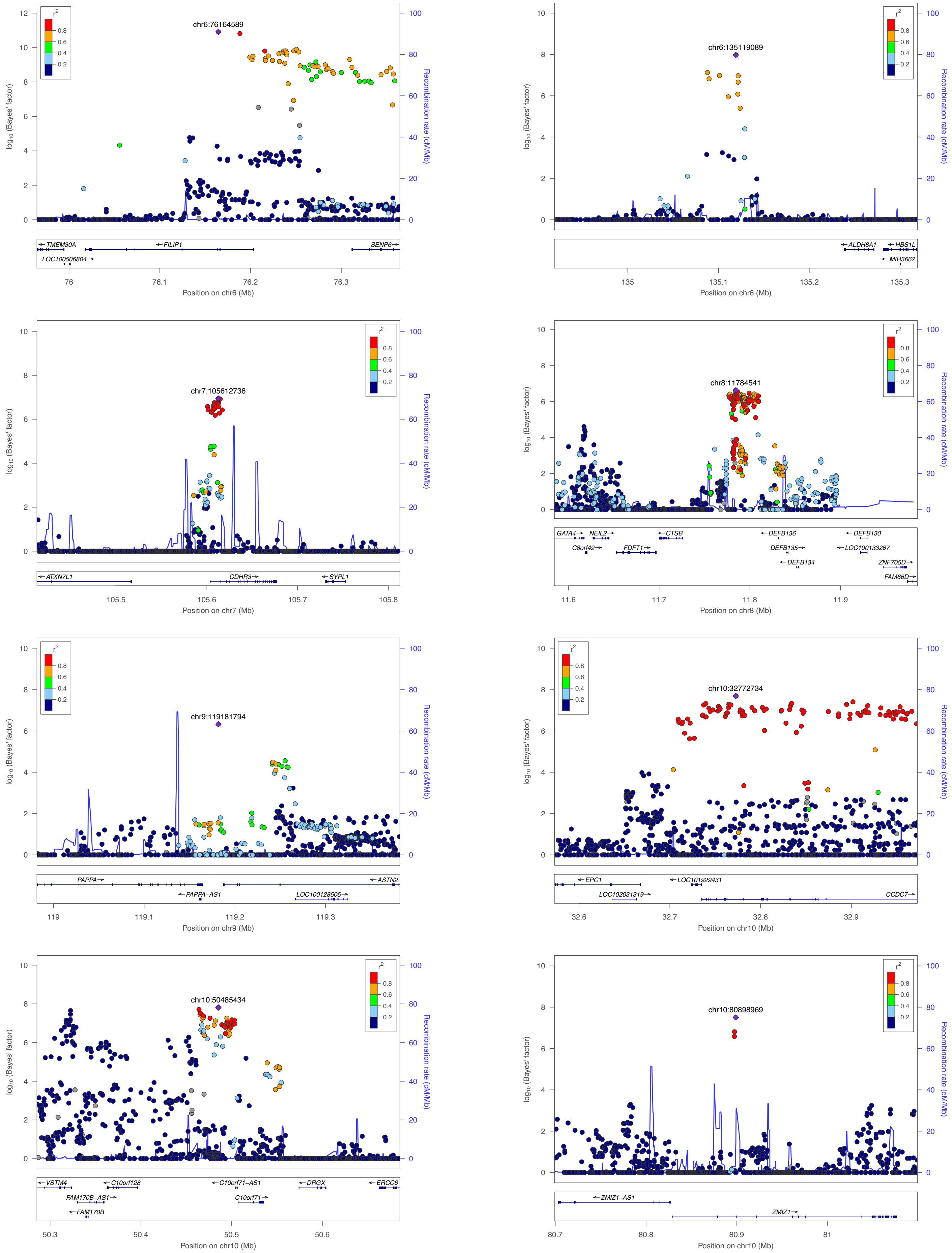

chr6:76164589
log10 (Bayes' factor)
Recombination rate (cM/Mb)
r2
TMEM30A
FILIP1
SENP6
LOC100506804
Position on chr6 (Mb)
chr6:135119089
ALDH8A1
HBS1L
MIR3662
Position on chr6 (Mb)
chr7:105612736
ATXN7L1
CDHR3
SYPL1
Position on chr7 (Mb)
chr8:11784541
GATA4
NEIL2
CTSB
DEFB136
DEFB130
C8orf49
FDFT1
DEFB135
LOC100133267
DEFB134
ZNF705D
FAM66D
Position on chr8 (Mb)
chr9:119181794
PAPPA
ASTN2
PAPPA-AS1
LOC100128505
Position on chr9 (Mb)
chr10:32772734
EPC1
LOC101929431
LOC102031319
CCDC7
Position on chr10 (Mb)
chr10:50485434
VSTM4
C10orf128
C10orf71-AS1
DRGX
ERCC6
FAM170B-AS1
C10orf71
FAM170B
Position on chr10 (Mb)
chr10:80898969
ZMIZ1-AS1
ZMIZ1
Position on chr10 (Mb)

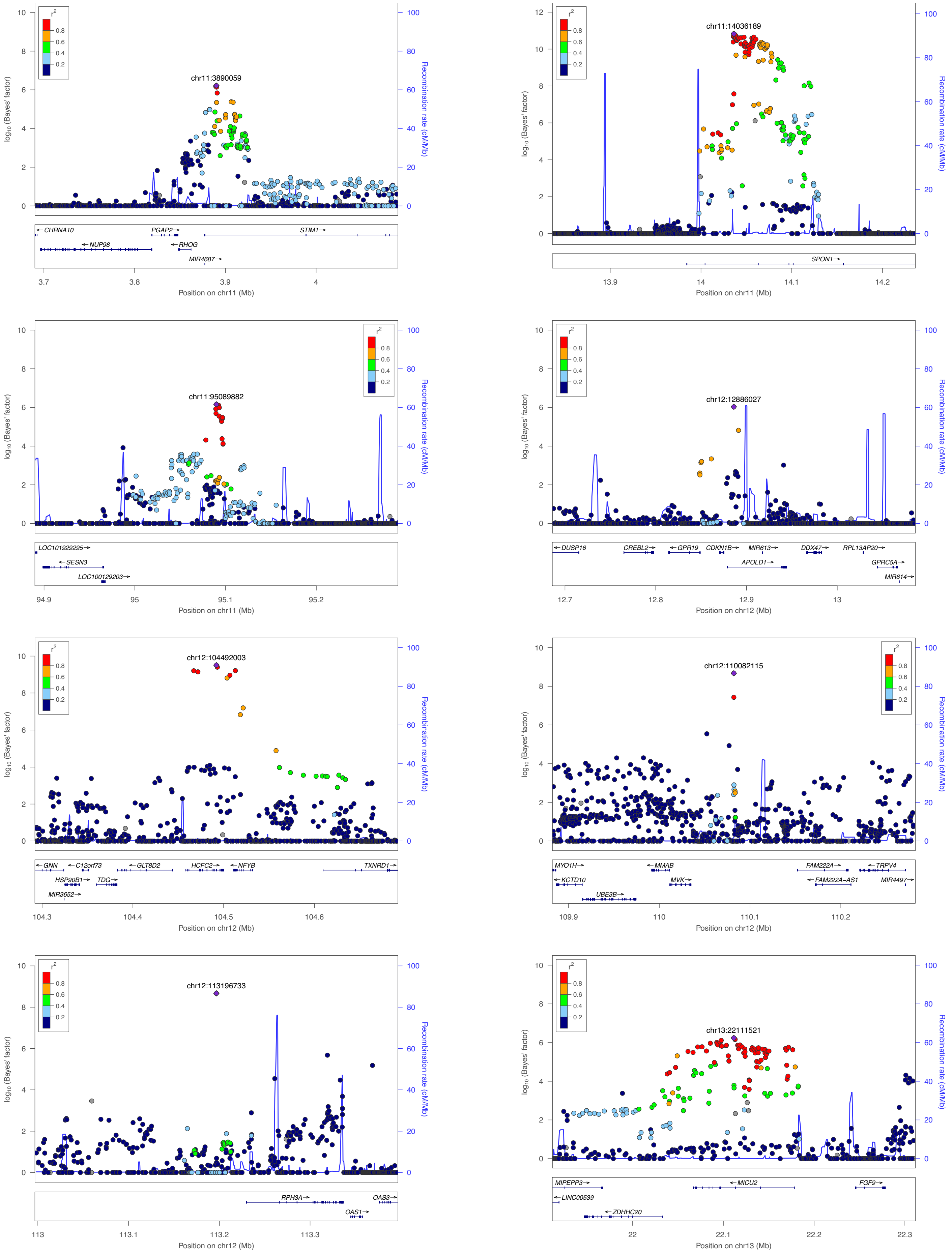

chr11:3890059
CHRNA10
NUP98
PGAP2
RHOG
MIR4687
STIM1
Position on chr11 (Mb)
chr11:14036189
SPON1
Position on chr11 (Mb)
chr11:95089882
LOC101929295
SESN3
LOC100129203
Position on chr11 (Mb)
chr12:12886027
DUSP16
CREBL2
GPR19
CDKN1B
APOLD1
MIR613
DDX47
RPL13AP20
GPRC5A
MIR614
Position on chr12 (Mb)
chr12:104492003
GNN
HSP90B1
MIR3652
C12orf73
TDG
GLT8D2
HCFC2
NFYB
TXNRD1
Position on chr12 (Mb)
chr12:110082115
MYO1H
KCTD10
UBE3B
MMAB
MVK
FAM222A
FAM222A-AS1
TRPV4
MIR4497
Position on chr12 (Mb)
chr12:113196733
RPH3A
OAS1
OAS3
Position on chr12 (Mb)
chr13:22111521
MIPEPP3
LINC00539
ZDHHC20
MICU2
FGF9
Position on chr13 (Mb)
log10 (Bayes' factor)
Recombination rate (cM/Mb)
r2

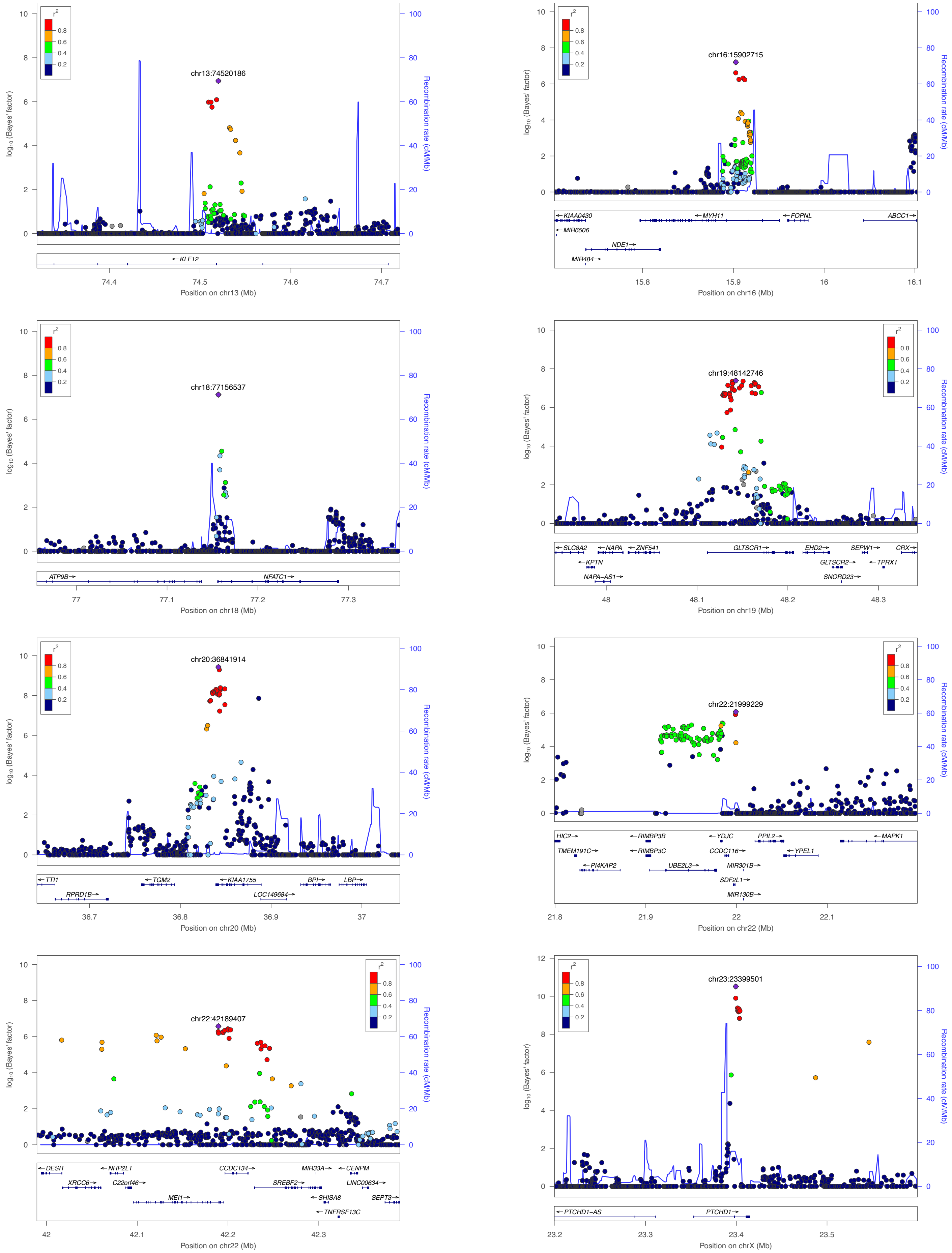

chr13:74520186
log10 (Bayes' factor)
Recombination rate (cM/Mb)
r2
0.8
0.6
0.4
0.2
KLF12
Position on chr13 (Mb)
chr16:15902715
KIAA0430
MIR6506
NDE1
MIR484
MYH11
FOPNL
ABCC1
Position on chr16 (Mb)
chr18:77156537
ATP9B
NFATC1
Position on chr18 (Mb)
chr19:48142746
SLC8A2
KPTN
NAPA-AS1
NAPA
ZNF541
GLTSCR1
EHD2
GLTSCR2
SNORD23
SEPW1
TPRX1
CRX
Position on chr19 (Mb)
chr20:36841914
TTI1
RPRD1B
TGM2
KIAA1755
LOC149684
BPI
LBP
Position on chr20 (Mb)
chr22:21999229
HIC2
TMEM191C
PI4KAP2
RIMBP3B
RIMBP3C
UBE2L3
YDJC
CCDC116
MIR301B
SDF2L1
MIR130B
PPIL2
YPEL1
MAPK1
Position on chr22 (Mb)
chr22:42189407
DESI1
XRCC6
NHP2L1
C22orf46
MEI1
CCDC134
SREBF2
MIR33A
SHISA8
TNFRSF13C
CENPM
LINC00634
SEPT3
Position on chr22 (Mb)
chr23:23399501
PTCHD1-AS
PTCHD1
Position on chrX (Mb)

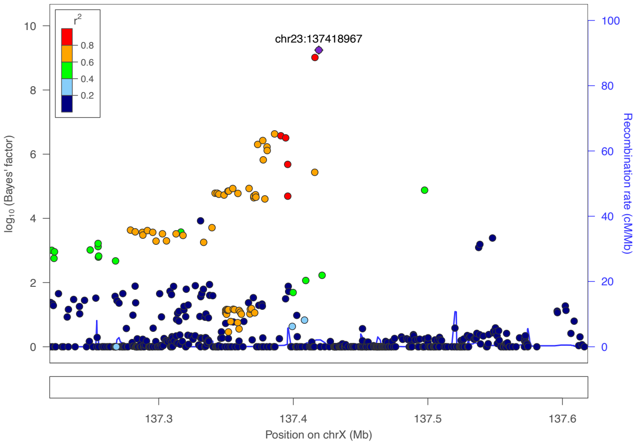

chr23:137418967
r2
0.8
0.6
0.4
0.2
log10 (Bayes' factor)
Recombination rate (cM/Mb)
Position on chrX (Mb)
137.3
137.4
137.5
137.6

4. Previously reported 127 loci in the trans-ancestry meta-GWAS

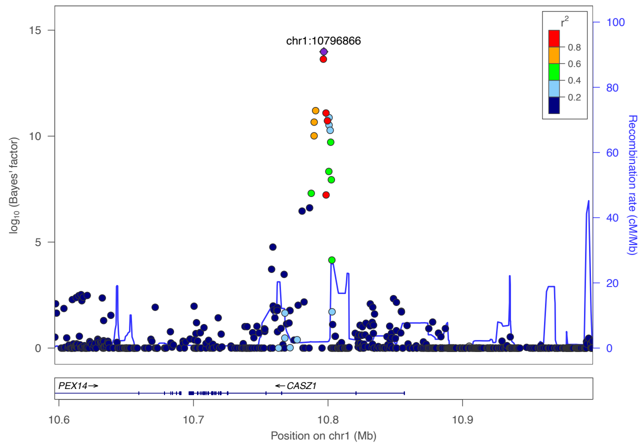

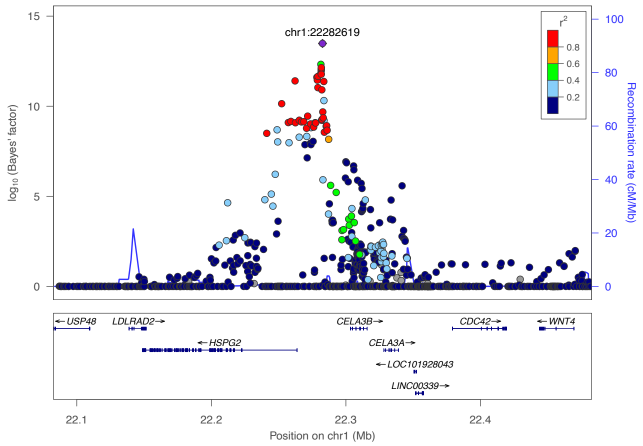

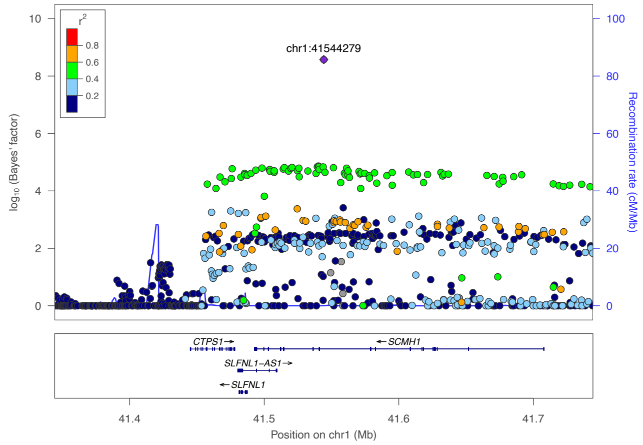

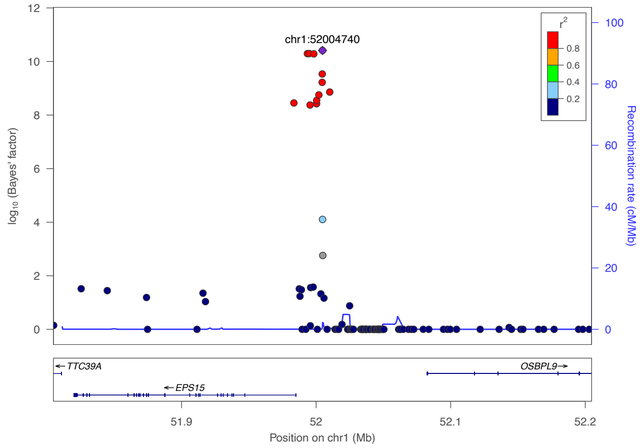

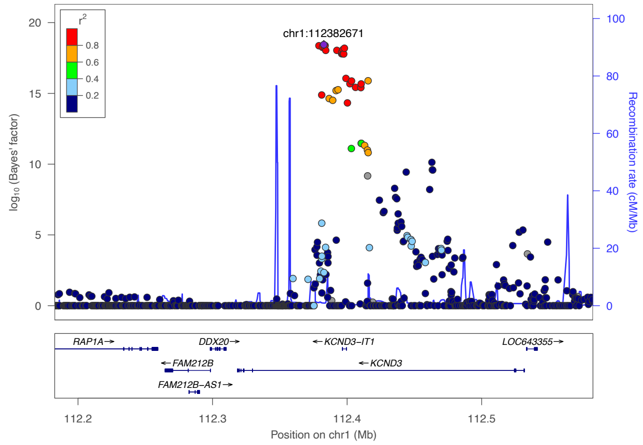

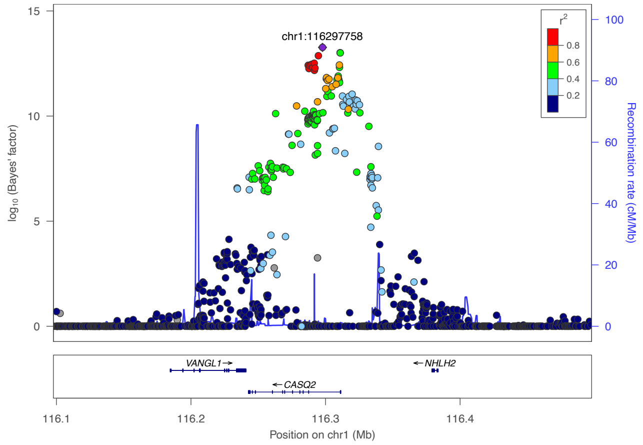

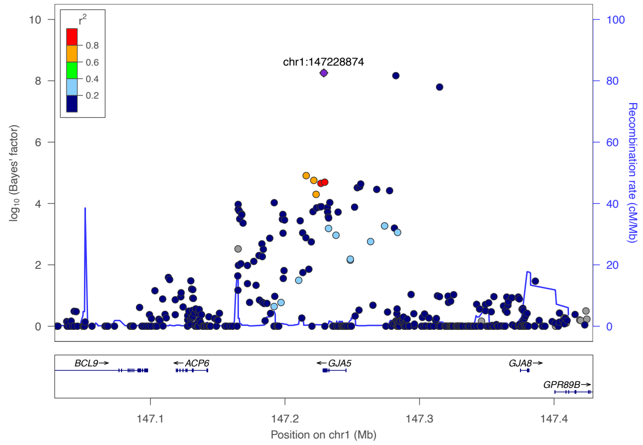

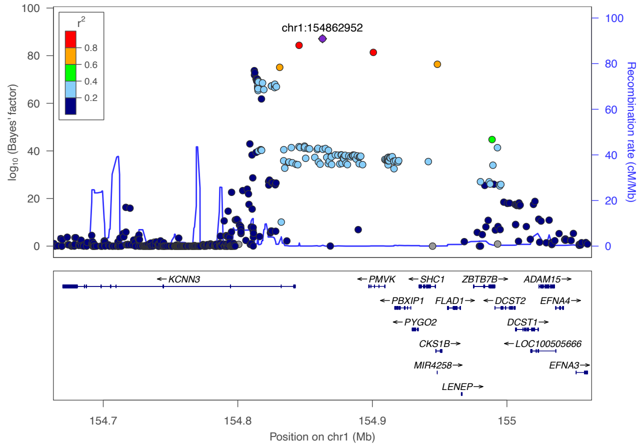

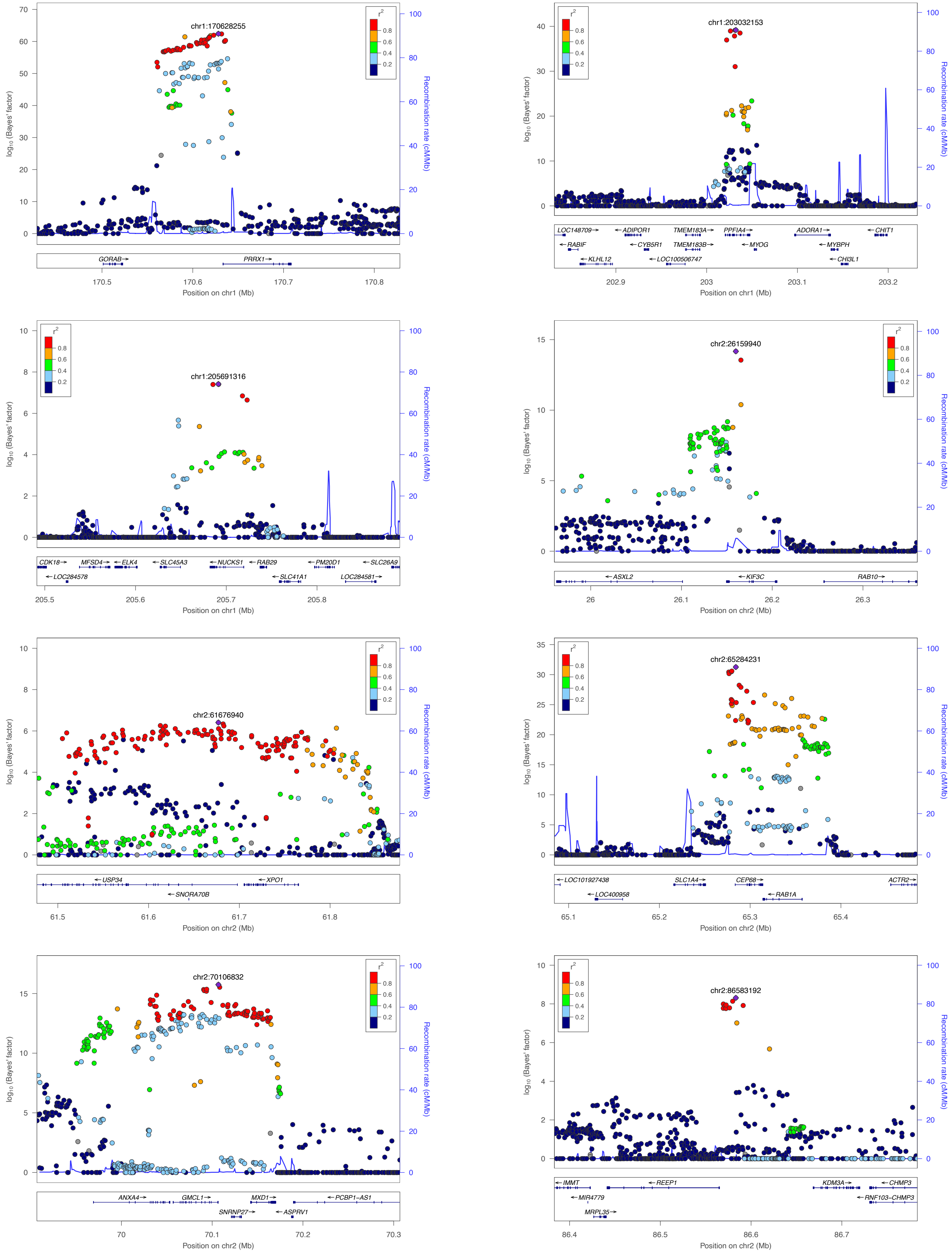

chr1:170628255
log10 (Bayes' factor)
Recombination rate (cM/Mb)
r2
0.8
0.6
0.4
0.2
GORAB→
PRRX1→
170.5
170.6
170.7
170.8
Position on chr1 (Mb)
chr1:203032153
LOC148709→
←ADIPOR1
TMEM183A→
PPFIA4→
ADORA1→
←CHIT1
←RABIF
←CYB5R1
TMEM183B→
←MYOG
←MYBPH
←KLHL12
←LOC100506747
←CHI3L1
202.9
203
203.1
203.2
Position on chr1 (Mb)
chr1:205691316
CDK18→
MFSD4→
←ELK4
←SLC45A3
←NUCKS1
←RAB29
←PM20D1
←SLC26A9
←LOC284578
←SLC41A1
LOC284581→
205.5
205.6
205.7
205.8
Position on chr1 (Mb)
chr2:26159940
←ASXL2
←KIF3C
RAB10→
26
26.1
26.2
26.3
Position on chr2 (Mb)
chr2:61676940
←USP34
←XPO1
←SNORA70B
61.5
61.6
61.7
61.8
Position on chr2 (Mb)
chr2:65284231
←LOC101927438
SLC1A4→
CEP68→
ACTR2→
←LOC400958
←RAB1A
65.1
65.2
65.3
65.4
Position on chr2 (Mb)
chr2:70106832
ANXA4→
GMCL1→
MXD1→
←PCBP1−AS1
SNRNP27→
←ASPRV1
70
70.1
70.2
70.3
Position on chr2 (Mb)
chr2:86583192
←IMMT
←REEP1
KDM3A→
←CHMP3
←MIR4779
←RNF103−CHMP3
MRPL35→
86.4
86.5
86.6
86.7
Position on chr2 (Mb)

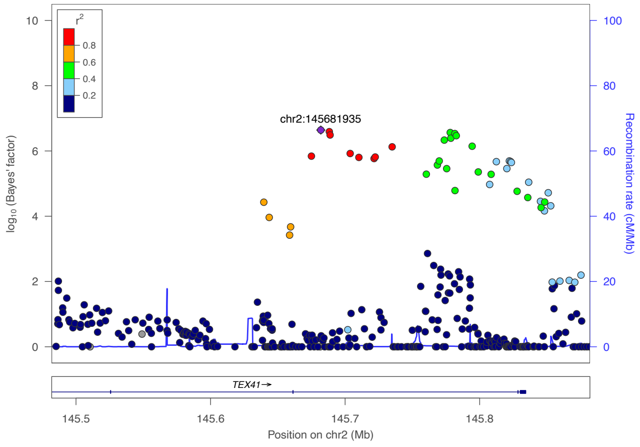
chr2:145681935
log10 (Bayes' factor)
Recombination rate (cM/Mb)
r2
0.8
0.6
0.4
0.2
TEX41→
145.5
145.6
145.7
145.8
Position on chr2 (Mb)

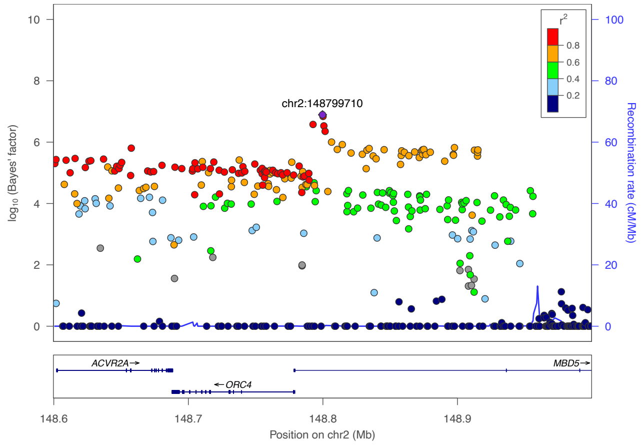
chr2:148799710
log10 (Bayes' factor)
Recombination rate (cM/Mb)
ACVR2A→
MBD5→
←ORC4
148.6
148.7
148.8
148.9
Position on chr2 (Mb)

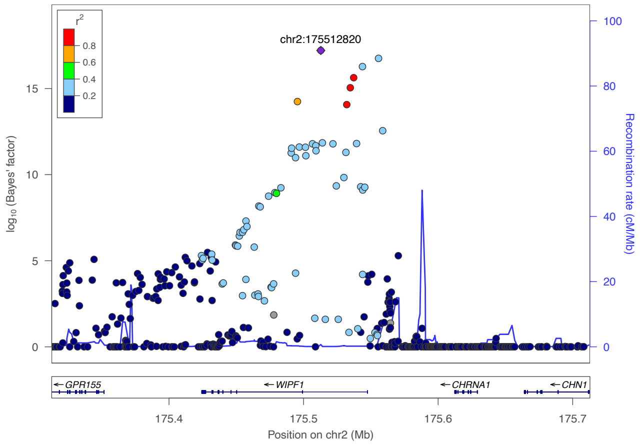
chr2:175512820
log10 (Bayes' factor)
Recombination rate (cM/Mb)
←GPR155
←WIPF1
←CHRNA1
←CHN1
175.4
175.5
175.6
175.7
Position on chr2 (Mb)

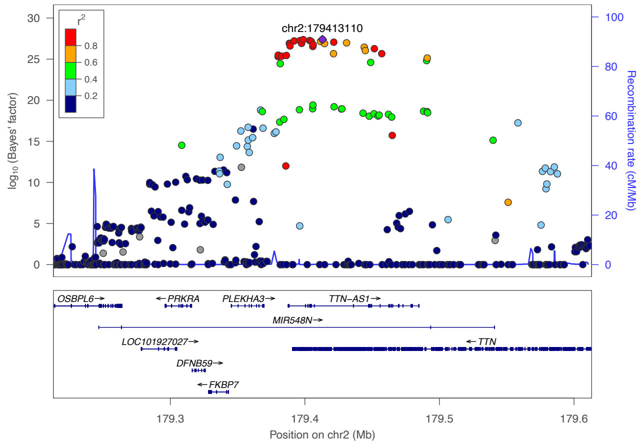
chr2:179413110
log10 (Bayes' factor)
Recombination rate (cM/Mb)
OSBPL6→
←PRKRA
PLEKHA3→
TTN−AS1→
MIR548N→
LOC101927027→
←TTN
DFNB59→
←FKBP7
179.3
179.4
179.5
179.6
Position on chr2 (Mb)

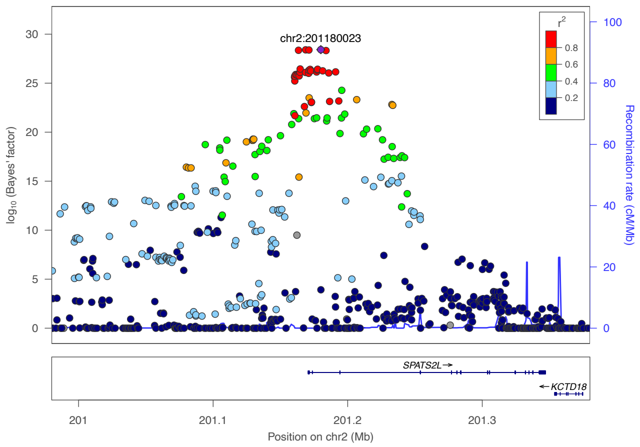
chr2:201180023
log10 (Bayes' factor)
Recombination rate (cM/Mb)
SPATS2L→
←KCTD18
201
201.1
201.2
201.3
Position on chr2 (Mb)

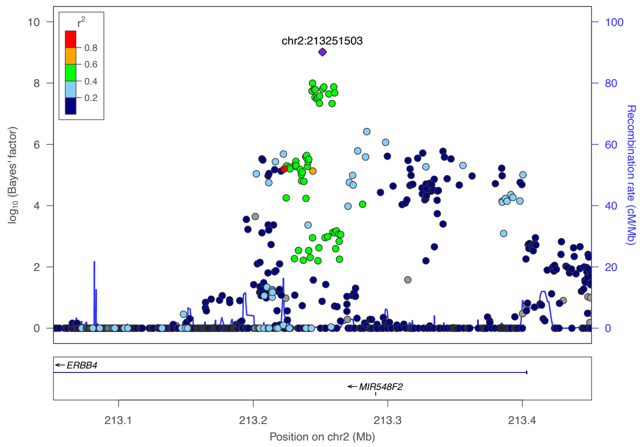
chr2:213251503
log10 (Bayes' factor)
Recombination rate (cM/Mb)
←ERBB4
←MIR548F2
213.1
213.2
213.3
213.4
Position on chr2 (Mb)

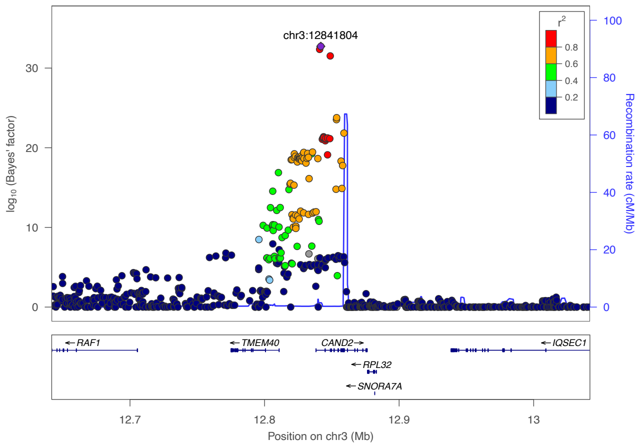
chr3:12841804
log10 (Bayes' factor)
Recombination rate (cM/Mb)
←RAF1
←TMEM40
CAND2→
←IQSEC1
←RPL32
←SNORA7A
12.7
12.8
12.9
13
Position on chr3 (Mb)

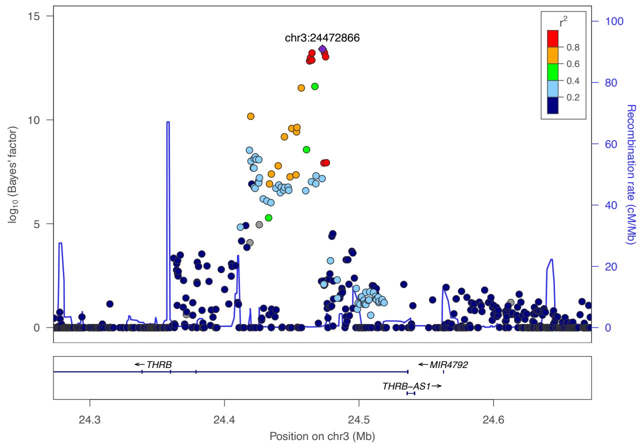
chr3:24472866
log10 (Bayes' factor)
Recombination rate (cM/Mb)
←THRB
←MIR4792
THRB−AS1→
24.3
24.4
24.5
24.6
Position on chr3 (Mb)

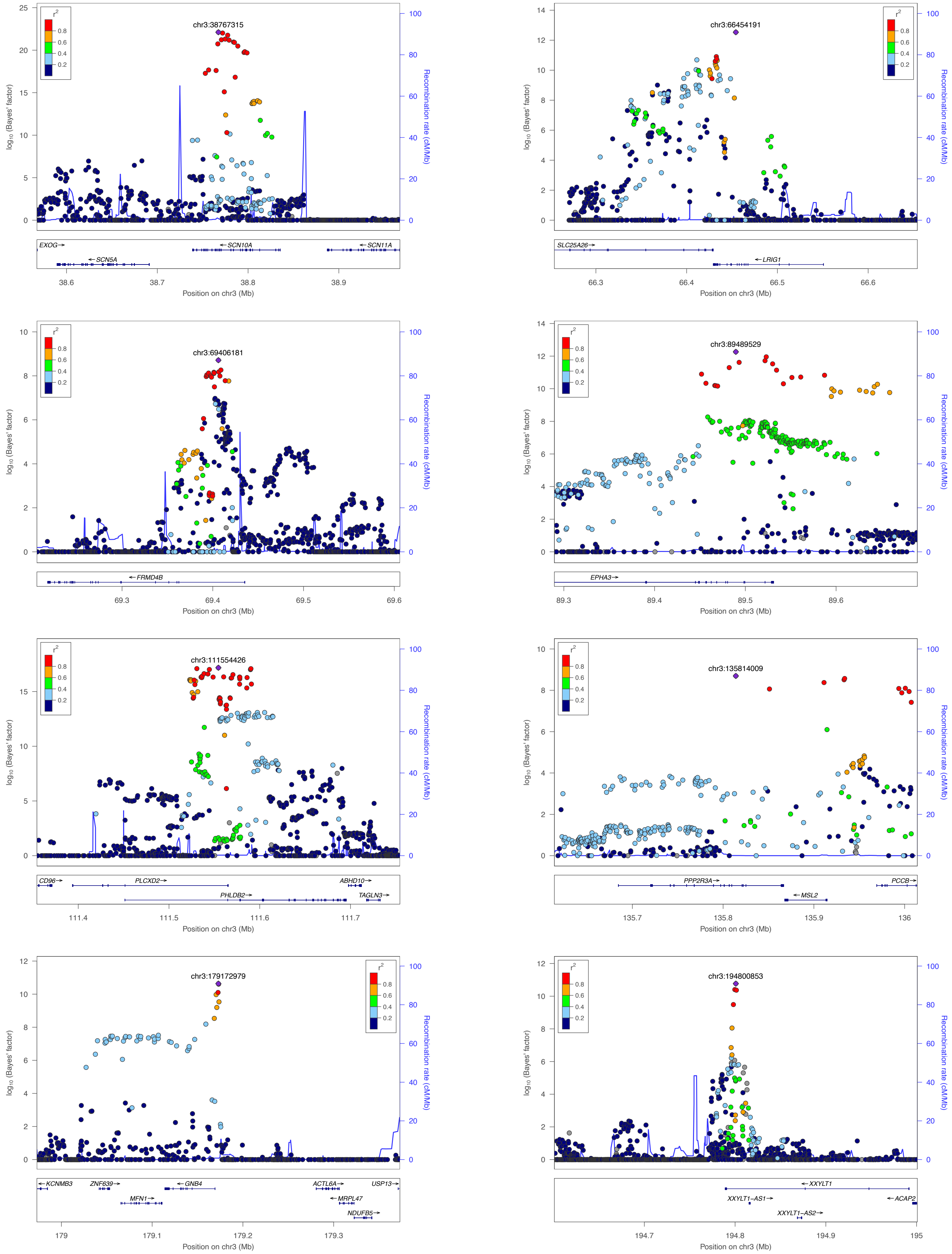

chr3:38767315
log10 (Bayes' factor)
Recombination rate (cM/Mb)
r2
EXOG
SCN5A
SCN10A
SCN11A
Position on chr3 (Mb)
chr3:66454191
SLC25A26
LRIG1
chr3:69406181
FRMD4B
chr3:89489529
EPHA3
chr3:111554426
CD96
PLCXD2
ABHD10
PHLDB2
TAGLN3
chr3:135814009
PPP2R3A
PCCB
MSL2
chr3:179172979
KCNMB3
ZNF639
GNB4
ACTL6A
USP13
MFN1
MRPL47
NDUFB5
chr3:194800853
XXYLT1
XXYLT1−AS1
ACAP2
XXYLT1−AS2

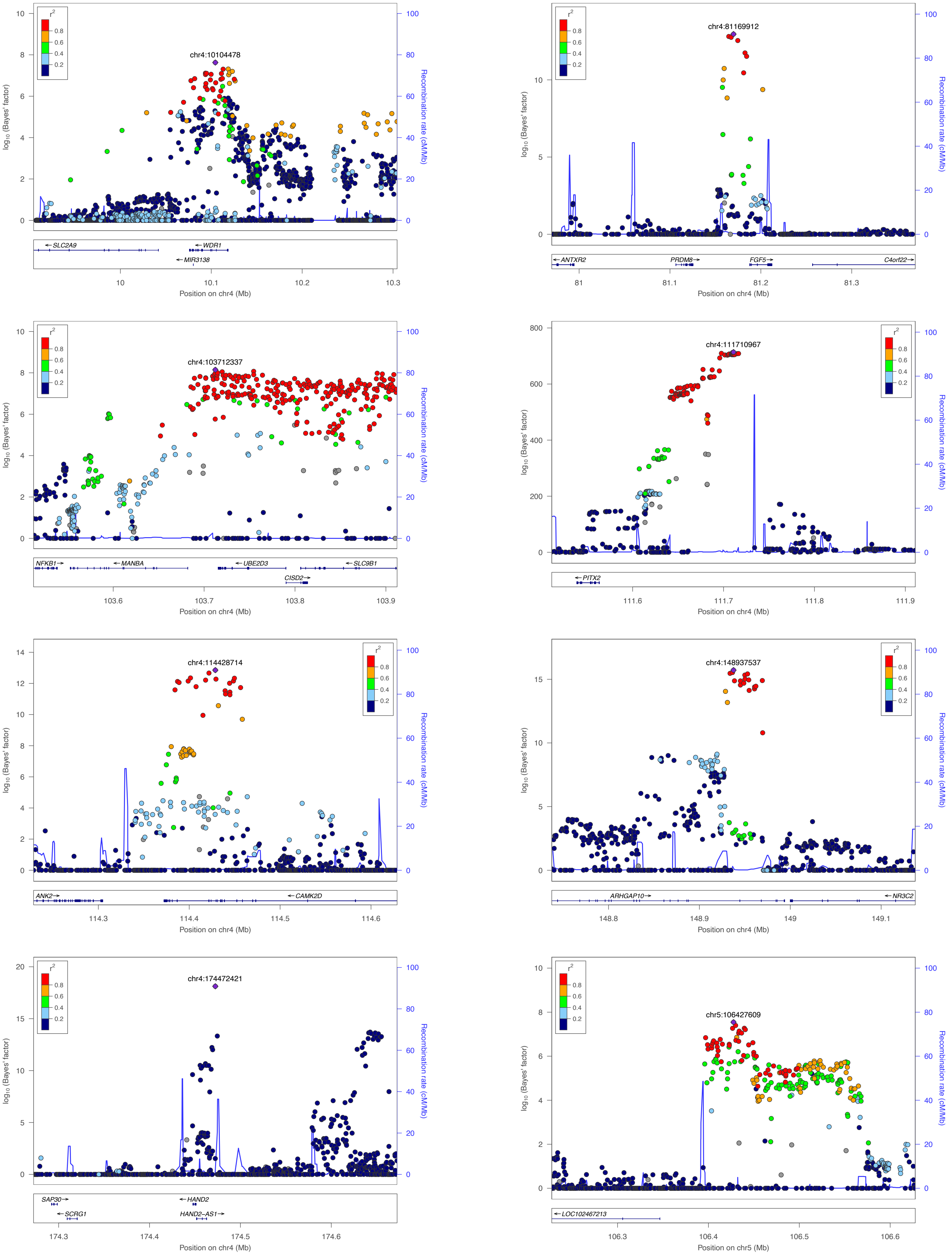
chr4:10104478
SLC2A9
WDR1
MIR3138
Position on chr4 (Mb)
chr4:81169912
ANTXR2
PRDM8
FGF5
C4orf22
Position on chr4 (Mb)
chr4:103712337
NFKB1
MANBA
UBE2D3
SLC9B1
CISD2
Position on chr4 (Mb)
chr4:111710967
PITX2
Position on chr4 (Mb)
chr4:114428714
ANK2
CAMK2D
Position on chr4 (Mb)
chr4:148937537
ARHGAP10
NR3C2
Position on chr4 (Mb)
chr4:174472421
SAP30
SCRG1
HAND2
HAND2−AS1
Position on chr4 (Mb)
chr5:106427609
LOC102467213
Position on chr5 (Mb)
log10 (Bayes' factor)
Recombination rate (cM/Mb)
r2
0.8
0.6
0.4
0.2

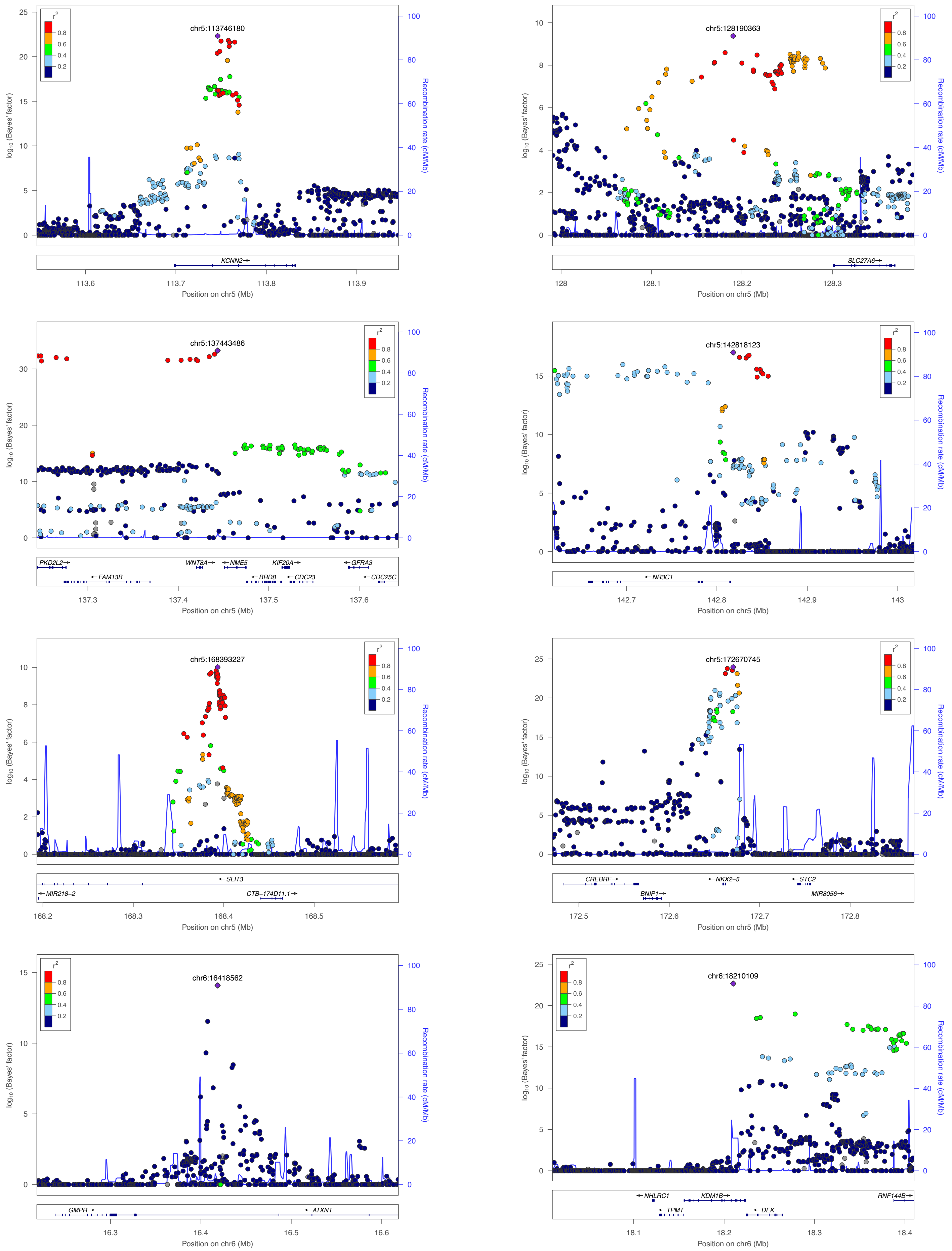

chr5:113746180
KCNN2→
Position on chr5 (Mb)
log10 (Bayes' factor)
Recombination rate (cM/Mb)
chr5:128190363
SLC27A6→
chr5:137443486
PKD2L2→
←FAM13B
WNT8A→
←NME5
←BRD8
KIF20A→
←CDC23
←GFRA3
←CDC25C
chr5:142818123
←NR3C1
chr5:168393227
←SLIT3
←MIR218−2
CTB−174D11.1→
chr5:172670745
CREBRF→
BNIP1→
←NKX2−5
←STC2
MIR8056→
chr6:16418562
GMPR→
←ATXN1
Position on chr6 (Mb)
chr6:18210109
←NHLRC1
KDM1B→
←TPMT
←DEK
RNF144B→

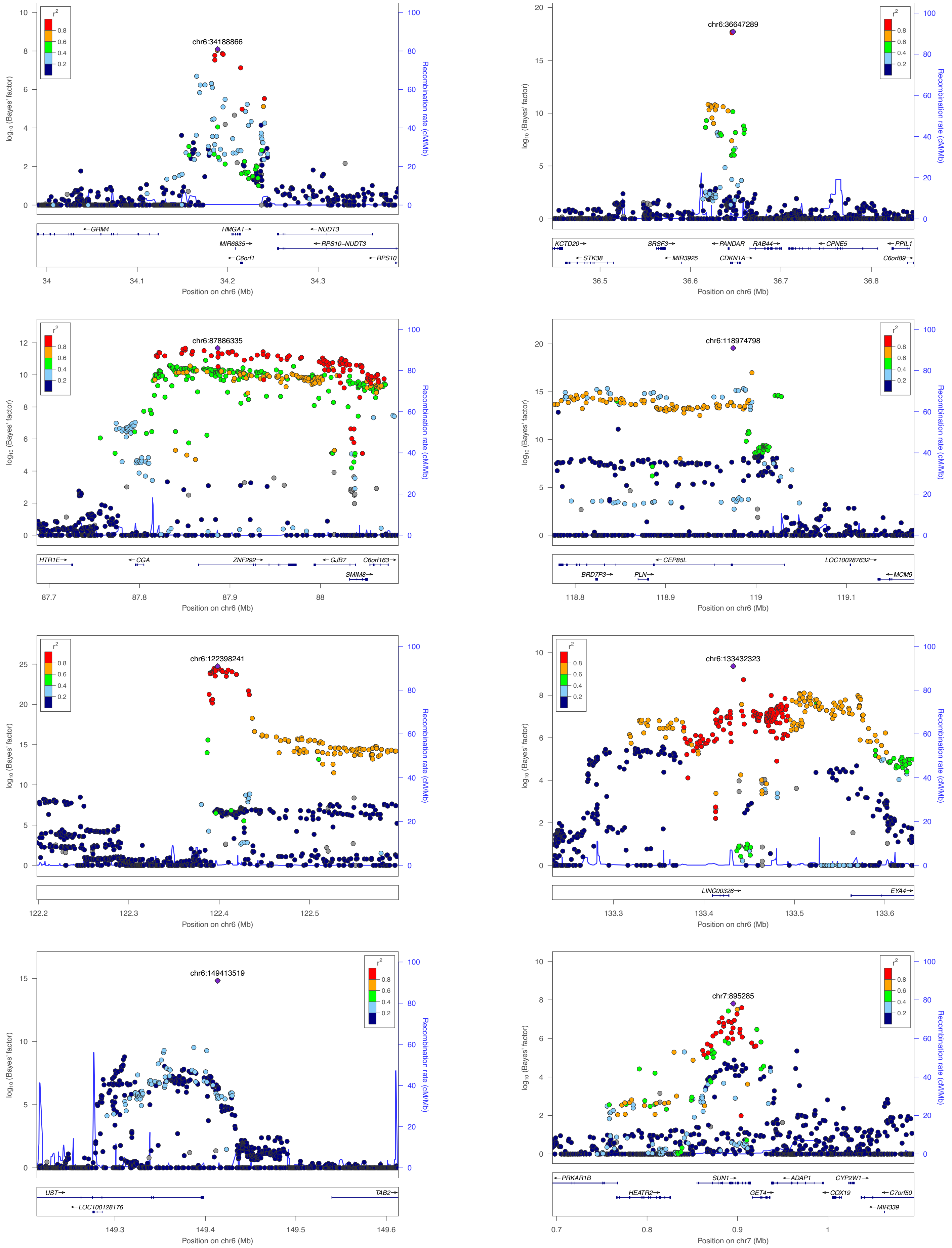
chr6:34188866
GRM4
HMGA1
NUDT3
MIR6835
RPS10−NUDT3
C6orf1
RPS10
Position on chr6 (Mb)
chr6:36647289
KCTD20
SRSF3
PANDAR
RAB44
CPNE5
PPIL1
STK38
MIR3925
CDKN1A
C6orf89
Position on chr6 (Mb)
chr6:87886335
HTR1E
CGA
ZNF292
GJB7
C6orf163
SMIM8
Position on chr6 (Mb)
chr6:118974798
CEP85L
LOC100287632
BRD7P3
PLN
MCM9
Position on chr6 (Mb)
chr6:122398241
Position on chr6 (Mb)
chr6:133432323
LINC00326
EYA4
Position on chr6 (Mb)
chr6:149413519
UST
TAB2
LOC100128176
Position on chr6 (Mb)
chr7:895285
PRKAR1B
SUN1
ADAP1
CYP2W1
HEATR2
GET4
COX19
C7orf50
MIR339
Position on chr7 (Mb)
$\log_{10}$ (Bayes' factor)
Recombination rate (cM/Mb)
r^2
0.8
0.6
0.4
0.2

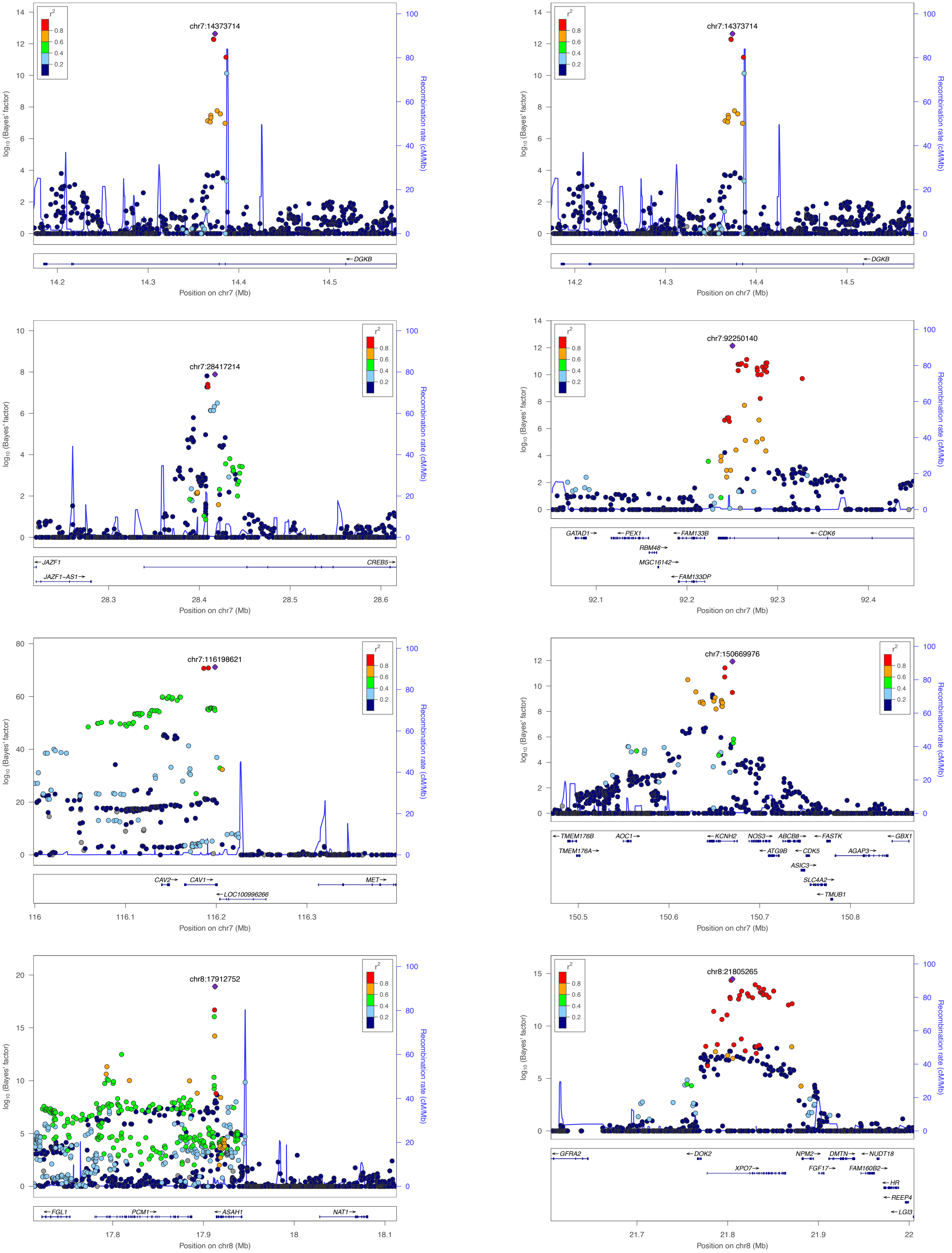

chr7:14373714
DGKB
Position on chr7 (Mb)
log10 (Bayes' factor)
Recombination rate (cM/Mb)
r2
chr7:14373714
DGKB
Position on chr7 (Mb)
chr7:28417214
JAZF1
JAZF1-AS1
CREB5
Position on chr7 (Mb)
chr7:92250140
GATAD1
PEX1
FAM133B
CDK6
RBM48
MGC16142
FAM133DP
Position on chr7 (Mb)
chr7:116198621
CAV2
CAV1
MET
LOC100996266
Position on chr7 (Mb)
chr7:150669976
TMEM176B
AOC1
KCNH2
NOS3
ABCB8
FASTK
GBX1
TMEM176A
ATG9B
CDK5
AGAP3
ASIC3
SLC4A2
TMUB1
Position on chr7 (Mb)
chr8:17912752
FGL1
PCM1
ASAH1
NAT1
Position on chr8 (Mb)
chr8:21805265
GFRA2
DOK2
NPM2
DMTN
NUDT18
XPO7
FGF17
FAM160B2
HR
REEP4
LGI3
Position on chr8 (Mb)

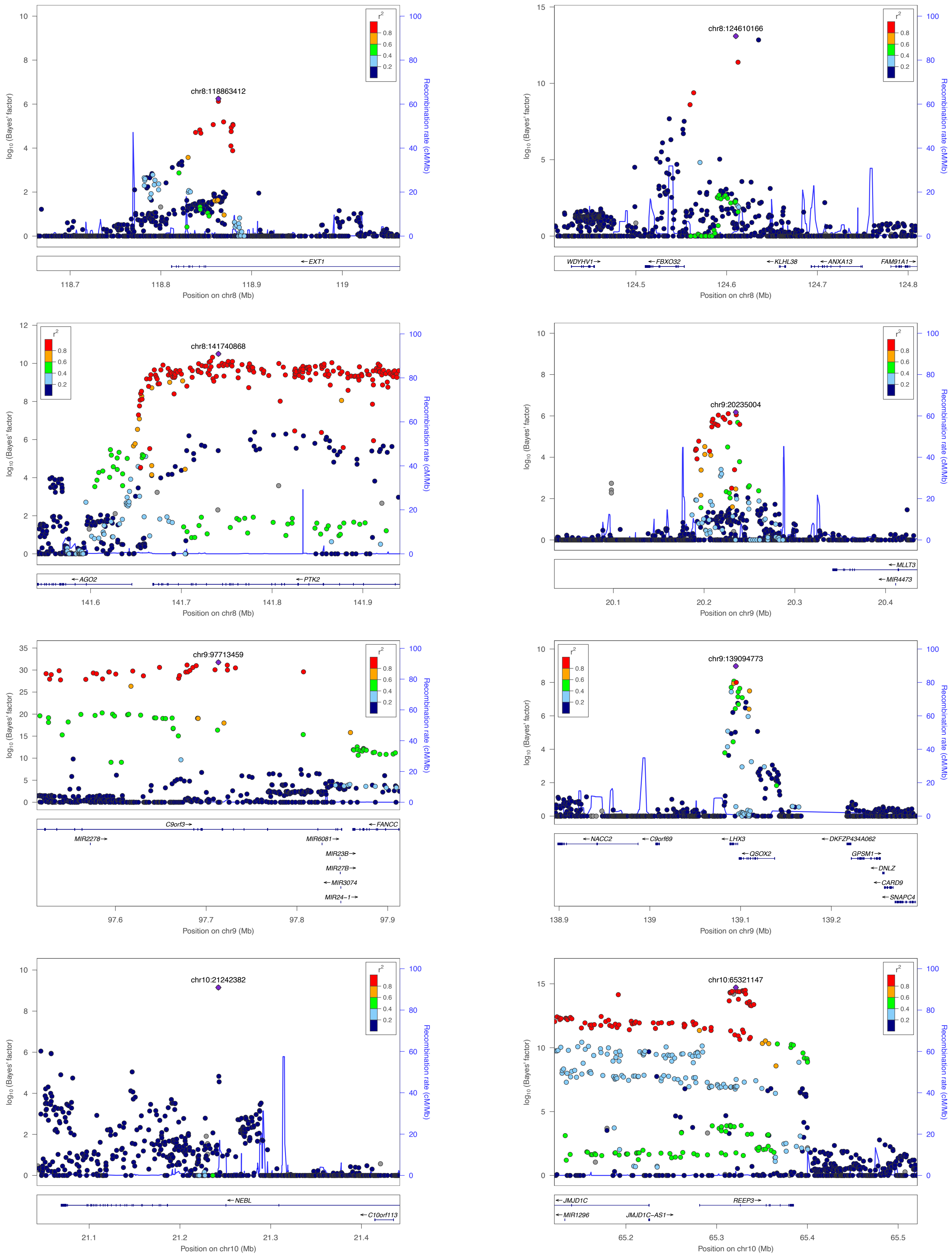

chr8:118863412
EXT1
Position on chr8 (Mb)
chr8:124610166
WDYHV1
FBXO32
KLHL38
ANXA13
FAM91A1
Position on chr8 (Mb)
chr8:141740868
AGO2
PTK2
Position on chr8 (Mb)
chr9:20235004
MLLT3
MIR4473
Position on chr9 (Mb)
chr9:97713459
C9orf3
FANCC
MIR2278
MIR6081
MIR23B
MIR27B
MIR3074
MIR24−1
Position on chr9 (Mb)
chr9:139094773
NACC2
C9orf69
LHX3
QSOX2
DKFZP434A062
GPSM1
DNLZ
CARD9
SNAPC4
Position on chr9 (Mb)
chr10:21242382
NEBL
C10orf113
Position on chr10 (Mb)
chr10:65321147
JMJD1C
REEP3
MIR1296
JMJD1C−AS1
Position on chr10 (Mb)
log10 (Bayes' factor)
Recombination rate (cM/Mb)
r2

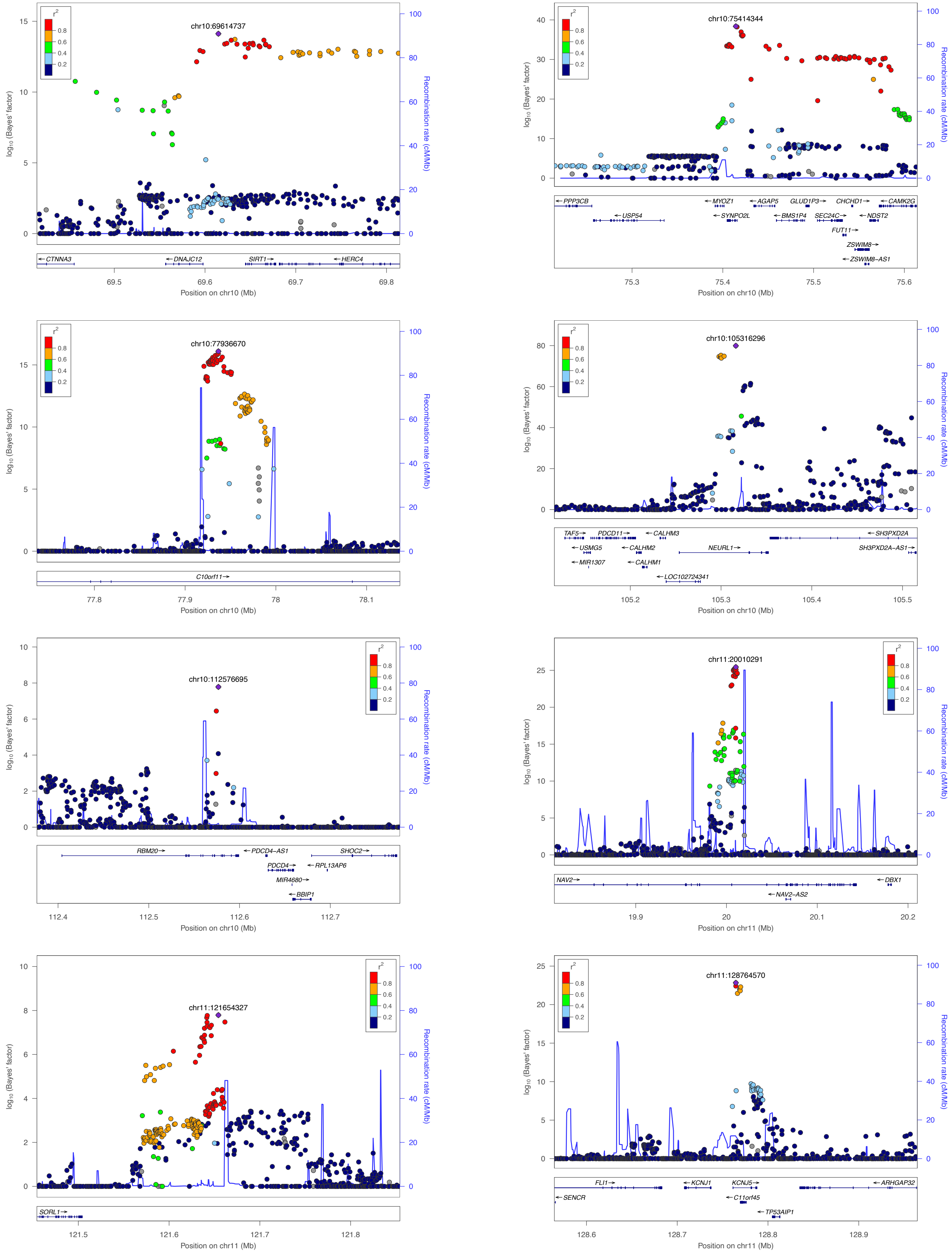

chr10:69614737
r²
log₁₀ (Bayes' factor)
Recombination rate (cM/Mb)
CTNNA3
DNAJC12
SIRT1
HERC4
Position on chr10 (Mb)
chr10:75414344
PPP3CB
USP54
MYOZ1
SYNPO2L
AGAP5
BMS1P4
GLUD1P3
SEC24C
CHCHD1
FUT11
NDST2
ZSWIM8
ZSWIM8-AS1
CAMK2G
Position on chr10 (Mb)
chr10:77936670
C10orf11
Position on chr10 (Mb)
chr10:105316296
TAF5
USMG5
MIR1307
PDCD11
CALHM2
CALHM1
CALHM3
LOC102724341
NEURL1
SH3PXD2A
SH3PXD2A-AS1
Position on chr10 (Mb)
chr10:112576695
RBM20
PDCD4-AS1
PDCD4
MIR4680
BBIP1
RPL13AP6
SHOC2
Position on chr10 (Mb)
chr11:20010291
NAV2
NAV2-AS2
DBX1
Position on chr11 (Mb)
chr11:121654327
SORL1
Position on chr11 (Mb)
chr11:128764570
FLI1
SENCR
KCNJ1
KCNJ5
C11orf45
TP53AIP1
ARHGAP32
Position on chr11 (Mb)

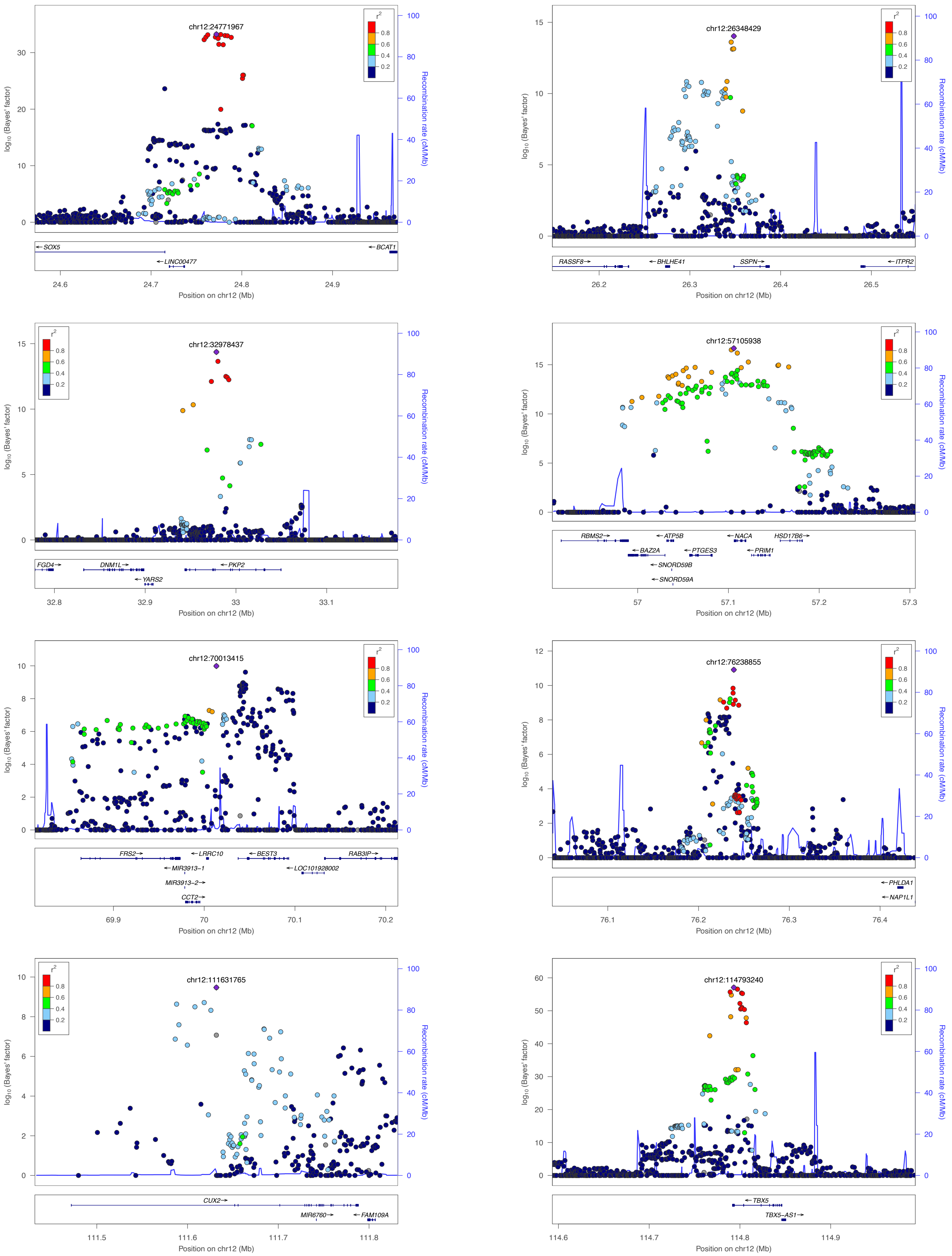
chr12:24771967
SOX5
LINC00477
BCAT1
chr12:26348429
RASSF8
BHLHE41
SSPN
ITPR2
chr12:32978437
FGD4
DNM1L
YARS2
PKP2
chr12:57105938
RBMS2
ATP5B
NACA
HSD17B6
BAZ2A
PTGES3
PRIM1
SNORD59B
SNORD59A
chr12:70013415
FRS2
LRRC10
BEST3
RAB3IP
MIR3913−1
LOC101928002
MIR3913−2
CCT2
chr12:76238855
PHLDA1
NAP1L1
chr12:111631765
CUX2
MIR6760
FAM109A
chr12:114793240
TBX5
TBX5−AS1
r²
log₁₀ (Bayes' factor)
Recombination rate (cM/Mb)
Position on chr12 (Mb)

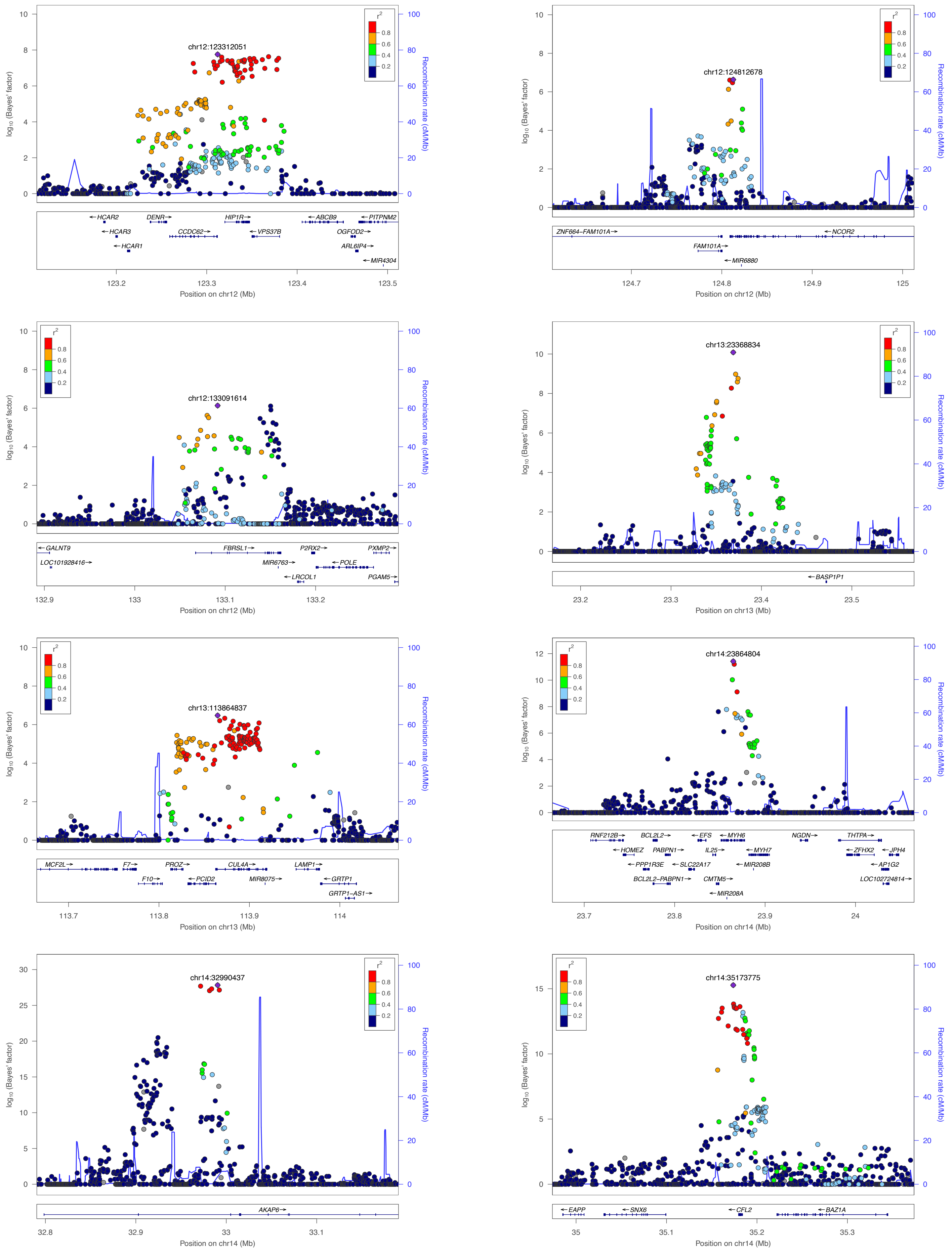

chr12:123312051
log10 (Bayes' factor)
Recombination rate (cM/Mb)
r2
0.8
0.6
0.4
0.2
HCAR2
HCAR3
HCAR1
DENR
CCDC62
HIP1R
VPS37B
ABCB9
OGFOD2
ARL6IP4
MIR4304
PITPNM2
123.2
123.3
123.4
123.5
Position on chr12 (Mb)
chr12:124812678
ZNF664−FAM101A
FAM101A
MIR6880
NCOR2
124.7
124.8
124.9
125
Position on chr12 (Mb)
chr12:133091614
GALNT9
LOC101928416
FBRSL1
MIR6763
LRCOL1
P2RX2
POLE
PXMP2
PGAM5
132.9
133
133.1
133.2
Position on chr12 (Mb)
chr13:23368834
BASP1P1
23.2
23.3
23.4
23.5
Position on chr13 (Mb)
chr13:113864837
MCF2L
F7
F10
PROZ
PCID2
CUL4A
MIR8075
LAMP1
GRTP1
GRTP1−AS1
113.7
113.8
113.9
114
Position on chr13 (Mb)
chr14:23864804
RNF212B
HOMEZ
PPP1R3E
BCL2L2
PABPN1
BCL2L2−PABPN1
SLC22A17
EFS
IL25
CMTM5
MYH6
MYH7
MIR208B
MIR208A
NGDN
THTPA
ZFHX2
JPH4
AP1G2
LOC102724814
23.7
23.8
23.9
24
Position on chr14 (Mb)
chr14:32990437
AKAP6
32.8
32.9
33
33.1
Position on chr14 (Mb)
chr14:35173775
EAPP
SNX6
CFL2
BAZ1A
35
35.1
35.2
35.3
Position on chr14 (Mb)

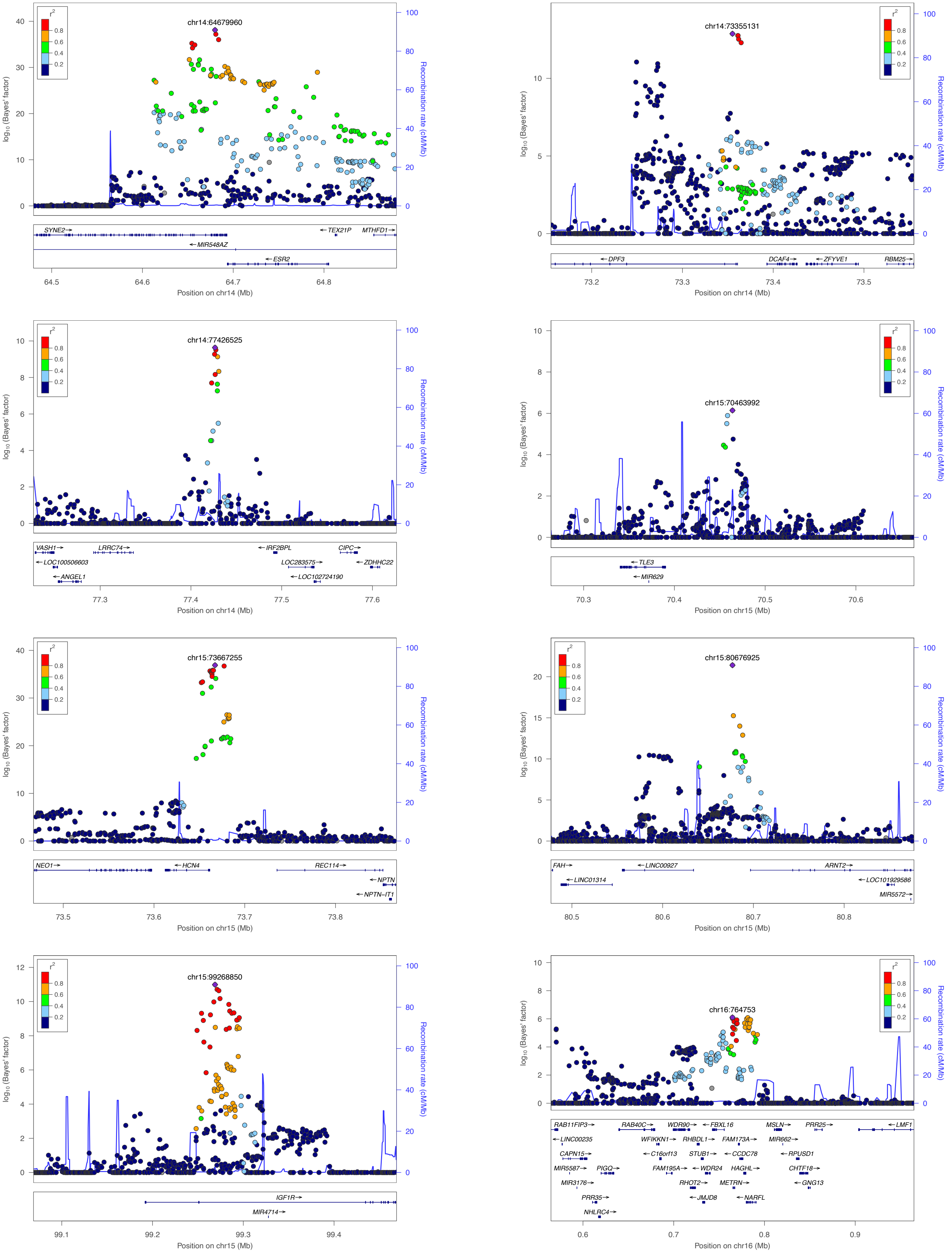

chr14:64679960
log10 (Bayes' factor)
Recombination rate (cM/Mb)
Position on chr14 (Mb)
SYNE2
TEX21P
MTHFD1
MIR548AZ
ESR2
chr14:73355131
DPF3
DCAF4
ZFYVE1
RBM25
chr14:77426525
VASH1
LRRC74
IRF2BPL
CIPC
LOC100506603
LOC283575
ZDHHC22
ANGEL1
LOC102724190
chr15:70463992
Position on chr15 (Mb)
TLE3
MIR629
chr15:73667255
NEO1
HCN4
REC114
NPTN
NPTN-IT1
chr15:80676925
FAH
LINC00927
ARNT2
LINC01314
LOC101929586
MIR5572
chr15:99268850
IGF1R
MIR4714
chr16:764753
Position on chr16 (Mb)
RAB11FIP3
RAB40C
WDR90
FBXL16
MSLN
PRR25
LMF1
LINC00235
WFIKKN1
RHBDL1
FAM173A
MIR662
CAPN15
C16orf13
STUB1
CCDC78
RPUSD1
MIR5587
PIGQ
FAM195A
WDR24
HAGHL
CHTF18
MIR3176
RHOT2
METRN
GNG13
PRR35
JMJD8
NARFL
NHLRC4

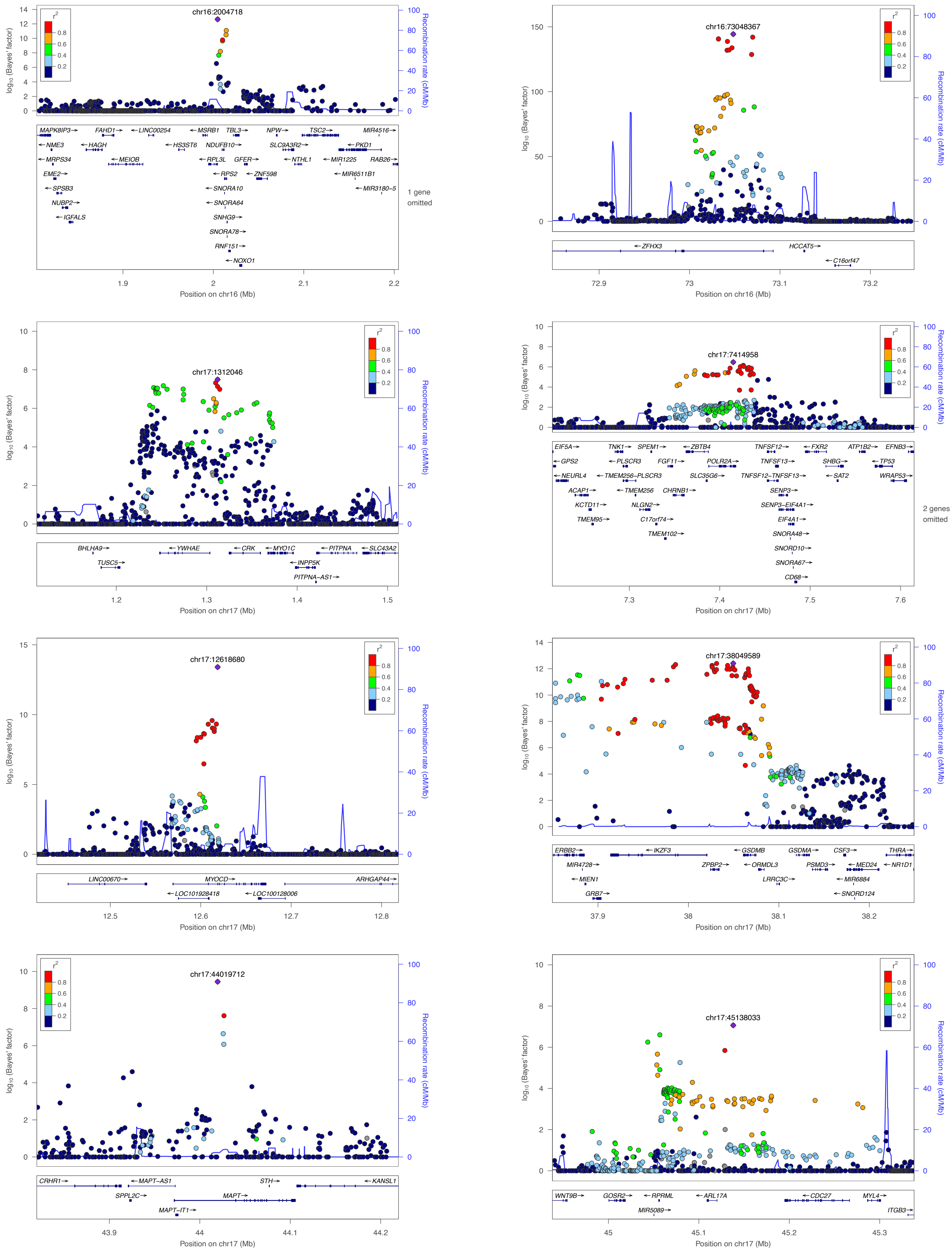
chr16:2004718
log10 (Bayes' factor)
Recombination rate (cM/Mb)
r2
0.8
0.6
0.4
0.2
MAPK8IP3
FAHD1
LINC00254
MSRB1
TBL3
NPW
TSC2
MIR4516
NME3
HAGH
HS3ST6
NDUFB10
SLC9A3R2
PKD1
MRPS34
MEIOB
RPL3L
GFER
NTHL1
MIR1225
RAB26
EME2
RPS2
ZNF598
MIR6511B1
SPSB3
SNORA10
MIR3180-5
NUBP2
SNORA64
IGFALS
SNHG9
SNORA78
RNF151
NOXO1
1 gene omitted
Position on chr16 (Mb)
chr16:73048367
ZFHX3
HCCAT5
C16orf47
Position on chr16 (Mb)
chr17:1312046
BHLHA9
YWHAE
CRK
MYO1C
PITPNA
SLC43A2
TUSC5
INPP5K
PITPNA-AS1
Position on chr17 (Mb)
chr17:7414958
EIF5A
TNK1
SPEM1
ZBTB4
TNFSF12
FXR2
ATP1B2
EFNB3
GPS2
PLSCR3
FGF11
POLR2A
TNFSF13
SHBG
TP53
NEURL4
TMEM256-PLSCR3
SLC35G6
TNFSF12-TNFSF13
SAT2
WRAP53
ACAP1
TMEM256
CHRNB1
SENP3
KCTD11
NLGN2
SENP3-EIF4A1
TMEM95
C17orf74
EIF4A1
TMEM102
SNORA48
SNORD10
SNORA67
CD68
2 genes omitted
Position on chr17 (Mb)
chr17:12618680
LINC00670
MYOCD
ARHGAP44
LOC101928418
LOC100128006
Position on chr17 (Mb)
chr17:38049589
ERBB2
IKZF3
GSDMB
GSDMA
CSF3
THRA
MIR4728
ZPBP2
ORMDL3
PSMD3
MED24
NR1D1
MIEN1
LRRC3C
MIR6884
GRB7
SNORD124
Position on chr17 (Mb)
chr17:44019712
CRHR1
MAPT-AS1
STH
KANSL1
SPPL2C
MAPT
MAPT-IT1
Position on chr17 (Mb)
chr17:45138033
WNT9B
GOSR2
RPRML
ARL17A
CDC27
MYL4
MIR5089
ITGB3
Position on chr17 (Mb)

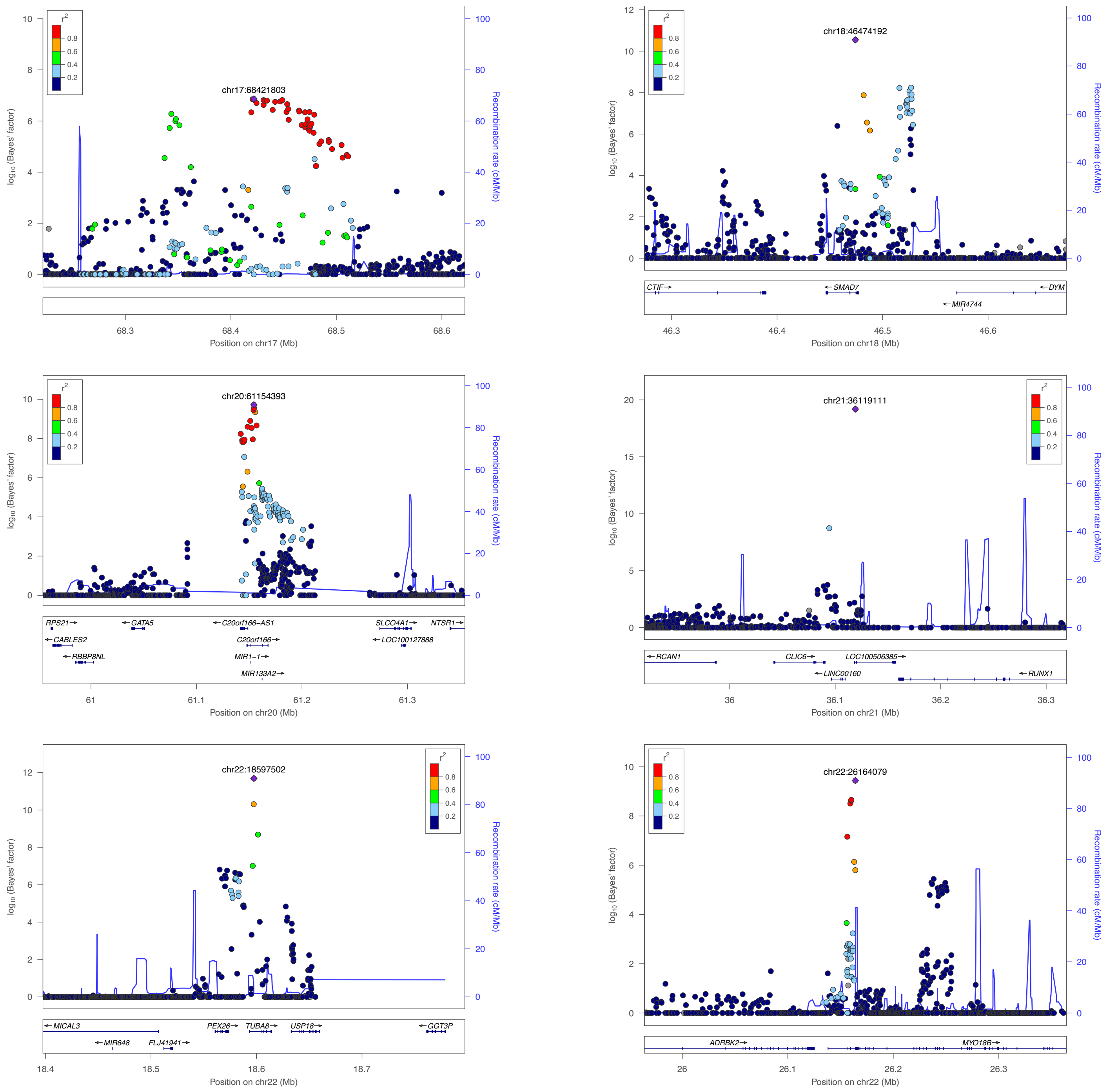

chr17:68421803
log10 (Bayes' factor)
Recombination rate (cM/Mb)
Position on chr17 (Mb)
r2
chr18:46474192
CTIF
SMAD7
DYM
MIR4744
Position on chr18 (Mb)
chr20:61154393
RPS21
GATA5
C20orf166−AS1
SLCO4A1
NTSR1
CABLES2
C20orf166
LOC100127888
RBBP8NL
MIR1−1
MIR133A2
Position on chr20 (Mb)
chr21:36119111
RCAN1
CLIC6
LOC100506385
LINC00160
RUNX1
Position on chr21 (Mb)
chr22:18597502
MICAL3
PEX26
TUBA8
USP18
GGT3P
MIR648
FLJ41941
Position on chr22 (Mb)
chr22:26164079
ADRBK2
MYO18B
Position on chr22 (Mb)
